# HSV-2 triggers upregulation of *MALAT1* in CD4^+^ T cells and promotes HIV latency reversal

**DOI:** 10.1172/JCI164317

**Published:** 2023-06-01

**Authors:** Carl A. Pierce, Lip Nam Loh, Holly R. Steach, Natalia Cheshenko, Paula Preston-Hurlburt, Fengrui Zhang, Stephanie Stransky, Leah Kravets, Simone Sidoli, William Philbrick, Michel Nassar, Smita Krishnaswamy, Kevan C. Herold, Betsy C. Herold

**Affiliations:** 1 Department of Microbiology and Immunology, Albert Einstein College of Medicine, New York, New York, USA.; 2 Department of Computational Biology,; 3 Department of Immunobiology, and; 4 Department of Medicine, Yale School of Medicine, New Haven, Connecticut, USA.; 5 Department of Biochemistry and; 6 Department of Otorhinolaryngology–Head and Neck Surgery, Albert Einstein College of Medicine, New York, New York, USA.; 7 Department of Genetics, Yale School of Medicine, New Haven, Connecticut, USA.; 8 Department of Pediatrics, Albert Einstein College of Medicine, New York, New York, USA.

**Keywords:** AIDS/HIV, Virology, T cells, Transcription

## Abstract

Herpes simplex virus type 2 (HSV-2) coinfection is associated with increased HIV-1 viral loads and expanded tissue reservoirs, but the mechanisms are not well defined. HSV-2 recurrences result in an influx of activated CD4^+^ T cells to sites of viral replication and an increase in activated CD4^+^ T cells in peripheral blood. We hypothesized that HSV-2 induces changes in these cells that facilitate HIV-1 reactivation and replication and tested this hypothesis in human CD4^+^ T cells and 2D10 cells, a model of HIV-1 latency. HSV-2 promoted latency reversal in HSV-2–infected and bystander 2D10 cells. Bulk and single-cell RNA-Seq studies of activated primary human CD4^+^ T cells identified decreased expression of HIV-1 restriction factors and increased expression of transcripts including *MALAT1* that could drive HIV replication in both the HSV-2–infected and bystander cells. Transfection of 2D10 cells with VP16, an HSV-2 protein that regulates transcription, significantly upregulated *MALAT1* expression, decreased trimethylation of lysine 27 on histone H3 protein, and triggered HIV latency reversal. Knockout of *MALAT1* from 2D10 cells abrogated the response to VP16 and reduced the response to HSV-2 infection. These results demonstrate that HSV-2 contributes to HIV-1 reactivation through diverse mechanisms, including upregulation of *MALAT1* to release epigenetic silencing.

## Introduction

Herpes simplex virus type 2 (HSV-2) is a common coinfection in persons living with human immunodeficiency virus type 1 (HIV-1) and is considered one of the most important cofactors driving the global HIV-1 epidemic. Epidemiologic studies consistently demonstrate that prevalent and incident HSV-2 infections are associated with an increased risk of HIV-1 acquisition and transmission (1). Moreover, among HIV-1/HSV-2–coinfected individuals, prevalent HSV-2 is associated with higher HIV-1 genital and plasma viral loads, which increase following HSV-2 outbreaks (2, 3). Subclinical shedding of HSV-2 is also associated with expanded HIV-1 tissue reservoirs and an increased divergence from the most recent common ancestor (4).

Most studies of the HIV-1 and HSV-2 syndemic have focused on local responses to HSV-2 that promote HIV-1 acquisition. For example, in HIV-1–seronegative (HIV^–^) individuals, symptomatic HSV-2 reactivation was associated with an influx of immune cells including activated CD4^+^ T cells at the site of lesions that may persist for a prolonged time (5). Even in the absence of clinical reactivation, an increase in activated CD4^+^, CCR5^+^ T cells in foreskin tissue and female genital tract samples and increased expression of T cell activation markers in the peripheral blood have been observed in HSV-2–seropositive (HSV-2^+^) compared with HSV-2–seronegative (HSV-2^–^) individuals (6, 7). These activated T cells could serve as targets for new HIV-1 infection but do not explain why coinfection is associated with an increased frequency of HIV-1 viremic episodes even among patients on antiretroviral therapy (8–10).

While asymptomatic HSV-2 infection was associated with the recruitment and persistence of activated CD4^+^ T cells in cervicovaginal samples obtained from HIV^–^ women, it was not associated with a significant difference in proinflammatory cytokines or chemokines in cervicovaginal fluid (6). Consistent with these observations, we also identified few significant differences in cytokine, chemokine, or antimicrobial peptide concentrations in genital tract secretions obtained from HIV-1^+^ women who were or were not coinfected with HSV-2 (11). However, we did find significant phenotypic differences in peripheral blood CD4^+^ (but not CD8^+^) T cells comparing coinfected versus HIV-1^+^, HSV-2^–^ women (12). Specifically, we found increased frequency of CCR5^+^, CXCR4^+^, PD-1^+^, and CD69^+^ and decreased frequency of CCR10^+^ and CCR6^+^ CD4^+^ T cells. These changes were associated with higher levels of cell-associated HIV-1 DNA. Paradoxically, IL-32, a proinflammatory cytokine, was lower in subpopulations of CD4^+^ T cells in HSV-2^+^ versus HSV-2^–^ women, and the addition of recombinant IL-32γ blocked HIV reactivation in CD4^+^ T cells treated with phytohemagglutinin (PHA) (12, 13). Other studies found that siRNA targeting IL-32 resulted in an increase in HIV replication (13). Together these findings suggested that the phenotypic changes in CD4^+^ T cells, including the decrease in IL-32γ associated with HSV-2, may promote HIV-1 reactivation and/or replication. However, the molecular mechanisms underlying these changes and their effects on HIV-1 reactivation are not known.

Activated CD4^+^ T cells are susceptible to HSV-2 infection in vitro (14), and virus has been detected in CD4^+^ T cells isolated from vesicle fluid of genital lesions and within biopsies of HSV-2 skin lesions (15). The recruitment and persistence of activated CD4^+^ T cells to the genital mucosa during HSV-2 reactivation (5) and the potential for activated peripheral blood CD4^+^ T cells to be exposed to HSV during episodes of transient HSV-2 viremia (16–18) prompted us to postulate that HSV-2 might have direct or indirect bystander effects on CD4^+^ T cells to promote HIV-1 reactivation and/or replication. We therefore analyzed the effects of HSV-2 infection of activated primary CD4^+^ T cells and an immortalized human CD4^+^ T cell line model of HIV-1 latency (Jurkat 2D10 cells) (19). We identified changes in the HSV-2–infected and bystander CD4^+^ T cells that were associated with HIV-1 reactivation, including upregulation of the long noncoding *MALAT1*. The ability of HSV-2 to trigger HIV latency reversal was reduced in *MALAT1*-knockout 2D10 cells.

## Results

### HSV-2 productively infects activated CD4^+^ T cells.

**Figure 1:**
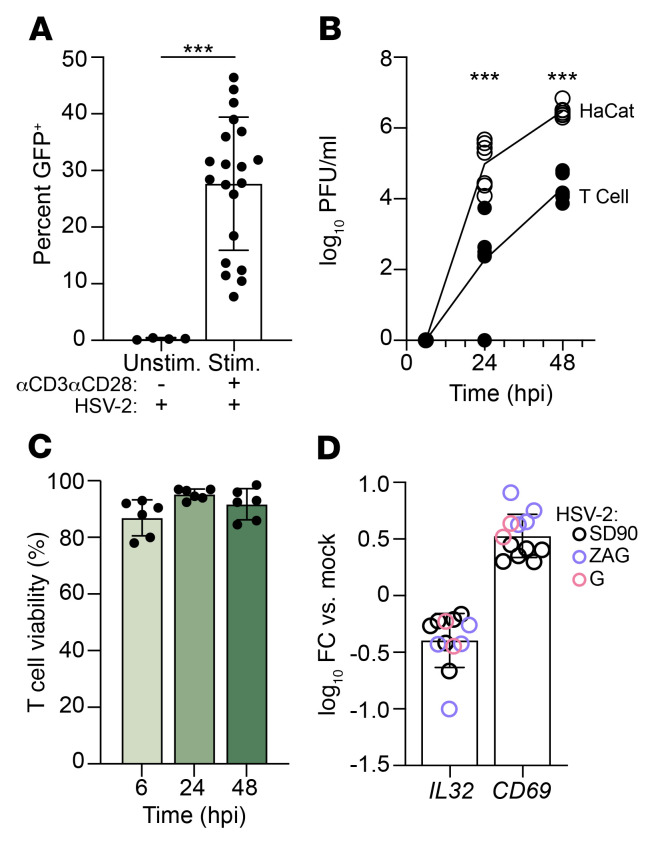
HSV-2 productively infects activated primary CD4^+^ T cells and downregulates IL-32 and upregulates CD69 expression. (**A**) Primary human CD4^+^ T cells isolated from healthy donor leukopaks were stimulated by CD3/CD28 cross-linking for 72 hours (*n* = 22) or left unstimulated (*n* = 4) and then incubated with GFP-expressing HSV-2(333ZAG) at an MOI of 1 PFU/cell for 2 hours, washed, and cultured for a further 22 hours, and the percentage of GFP^+^ cells was quantified by flow cytometry. (**B**) Primary anti-CD3/CD28–stimulated CD4^+^ T cells (filled symbols) or HaCaT cells (open symbols) were infected with HSV-2(SD90) (MOI = 0.001 PFU/cell), and at the indicated times, the amount of infectious virus released into the culture supernatants was quantified by plaque assays conducted in duplicate on Vero cells. Values of 0 PFU were set to zero before log transformation. Viral yields from the 2 different cell types were compared at 24 and 48 hours. ***P < 0.001, Mann-Whitney test. (**C**) CD4^+^ T cell viability following HSV-2 infection as in **B** was determined by vital dye exclusion (*n* = 6 donors). (**D**) Anti-CD3/CD28–stimulated CD4^+^ T cells from *n* = 3 different donors were infected with the indicated isolates of HSV-2 at an MOI of 1 PFU/cell, and at 24 hpi, *IL32* and *CD69* gene expression was quantified by RT-qPCR. Results are presented as log_10_ fold change (FC) relative to mock-infected CD4^+^ T cells.

To confirm the previously reported susceptibility of activated CD4^+^ T cells and Jurkat T cells to HSV-2 infection, we infected Jurkat or primary human CD4^+^ T cells with HSV-2(333ZAG), which expresses GFP under a CMV promoter (20). We observed a dose-dependent increase in the percentage of GFP^+^ Jurkat cells following exposure to a multiplicity of infection (MOI) of 1, 5, or 10 PFU/cell of 21.85%, 40.75%, and 45.90%, respectively (Supplemental Figure 1A; supplemental material available online with this article; https://doi.org/10.1172/JCI164317DS1). Exposure of stimulated (anti-CD3/anti-CD28) primary human CD4^+^ T cells (isolated from *n* = 20 different donor leukopaks) to HSV-2(333ZAG) (MOI = 1) resulted in 27.69% ± 11.79% (mean ± SD) GFP^+^ cells, whereas unstimulated cells were resistant (0.28% ± 0.16%) (Figure 1A and Supplemental Figure 1B). Productive HSV-2 infection of activated CD4^+^ T cells was confirmed using a low-passage clinical isolate, HSV-2(SD90), at MOI = 0.001 and quantification of infectious virus released into culture supernatants at 6, 24, and 48 hours post-infection (hpi) by plaque assay. Kinetics of infection in activated CD4^+^ T and HaCaT cells (human keratinocytes) were similar, although viral yields were lower (log_10_ PFU/mL = 6.40 vs. 4.12 at 48 hpi, *P* < 0.001) (Figure 1B). The mean CD4^+^ T cell viability was 86.92% (range 78.00%–93.00%) 6 hours, 95.25% (92.50%–97.00%) 24 hours, and 91.76% (84.50%–98.58%) 48 hours after infection (Figure 1C). Consistent with our previous observations, 24 hpi with 3 different isolates of HSV-2 (SD90, 333ZAG, or G) (MOI = 1), there was a 0.39 ± 0.24 log_10_-fold decrease in *IL32* and a 0.53 ± 0.19 log_10_-fold increase in *CD69* expression relative to mock-infected cells (*P* < 0.001) (Figure 1D) (12).

**Figure 2:**
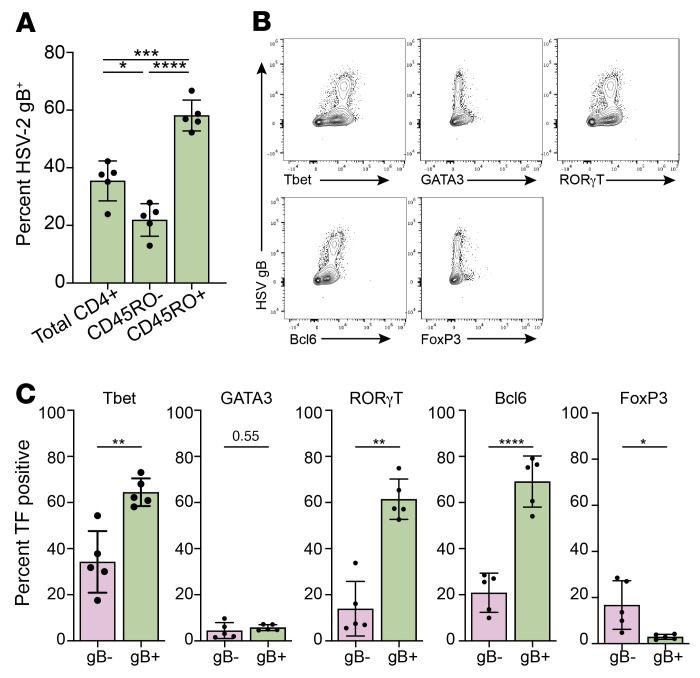
HSV-2–infected cells are preferentially CD45RO^+^ CD4^+^ T cells and express the transcription factors T-bet, RORγT, and Bcl6. (**A**) CD4^+^ T cells from *n* = 5 healthy donor leukopaks were stimulated for 72 hours by CD3/CD28 cross-linking, infected with HSV-2(SD90) (MOI = 1 PFU/cell), and cultured for a further 24 hours, and then stained for glycoprotein B (gB) and for CD45RO. The percentage of gB^+^ cells in the total CD4^+^ T cell (CD45RO^–/+^) population, CD4^+^CD45RO^–^ population, and CD4^+^CD45RO^+^ population was quantified by flow cytometry. *P < 0.05, ***P < 0.001, ****P < 0.0001, 1-way ANOVA. (**B**) Representative flow cytometry plots of HSV-2 gB (*y* axis) and transcription factor (*x* axis) staining with electronic gates placed on CD4^+^ T cells. (**C**) Cells were infected as in **A** and stained for gB and for the indicated transcription factors (TFs). The percentages of gB^+^ (infected) and gB^–^ (bystander) cells expressing the indicated markers were compared by paired *t* test; *P < 0.05, **P < 0.01, ****P < 0.0001.

We further characterized CD4^+^ T cells that were susceptible to HSV-2 infection by flow cytometry. Peripheral blood CD4^+^ T cells (stimulated with anti-CD3/CD28) were mock-infected or infected with HSV-2(SD90) (MOI = 1). Infected cells were identified by staining with a fluorophore-conjugated antibody against HSV-2 glycoprotein B (anti-gB). The gB^+^ cells were more likely to be CD45RO^+^ (58.13% ± 5.37% CD45RO^+^ vs. 21.90% ± 5.64% CD45RO^–^, mean ± SD, *P* < 0.0001) (Figure 2A) and to express Tbet (64.65% vs. 34.26%, *P* < 0.01), RORγT (61.47% vs. 13.97%, *P* < 0.01), and Bcl6 (69.09% vs. 20.91%, *P* < 0.0001) (Figure 2B). The frequency of GATA3^+^ cells was not different in the gB^+^ versus the gB^–^ cells (5.75% vs. 4.49%), and the frequency of FoxP3^+^ cells was lower (2.97% vs. 16.76%, *P* < 0.05) (Figure 2C).

### HSV-2 infection of CD4^+^ T cells promotes HIV reactivation and replication.

**Figure 3:**
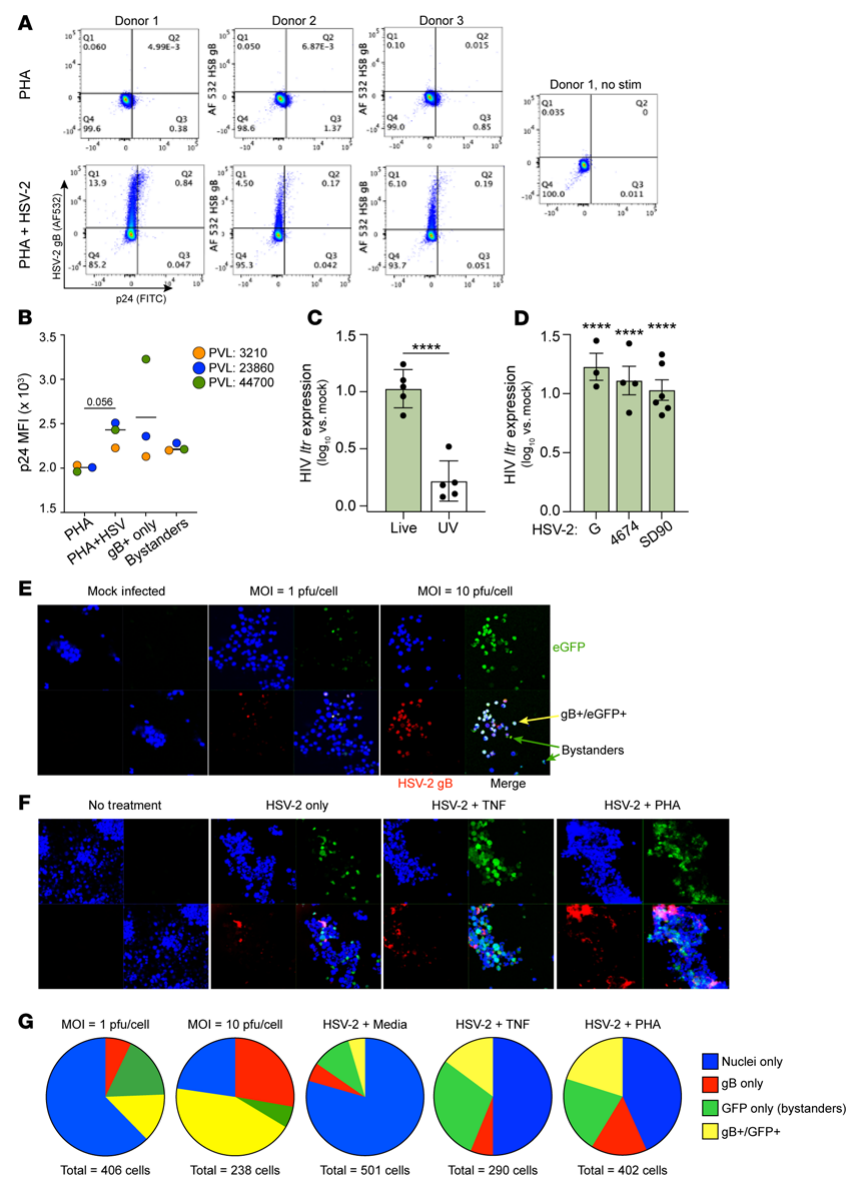
HSV-2 infection of T cells promotes HIV reactivation and replication. (**A** and **B**) CD4^+^ T cells from HIV^+^ donors with plasma viral loads (PVLs) of 3,210 (donor 1, orange), 23,860 (donor 2, blue), and 44,700 (donor 3, green) copies/mL, respectively, were stimulated with PHA for 24 hours and then mock-infected or infected with HSV-2(SD90) (MOI = 1 PFU/cell) and, 48 hpi, were stained for HSV-2 gB and HIV-1 p24 (**A**). The mean fluorescence intensity (MFI) of p24 was determined; the bar represents the median (*P* = 0.056, paired *t* test, PHA + HSV-2 vs. PHA alone) (**B**). (**C** and **D**) 2D10 cells were exposed to live or UV-inactivated (UV) HSV-2(G) (**C**) or to strain G, 4674, SD90, or heat-inactivated (HI) G (**D**) (MOI = 1 PFU/cell). HIV *ltr* gene expression was measured by RT-qPCR relative to mock-infected samples 24 hpi (*n* = 3–6 each, ****P < 0.0001 comparing live vs. UV in **C** [*t* test] and each strain versus HI virus by 1-way ANOVA in **D**). (**E**) 2D10 cells were infected with HSV-2(G) at an MOI of 1 or 10 PFU/cell, and at 24 hpi, cells were fixed and stained with anti-gB antibody (red). Nuclei were stained with DAPI (blue), and HIV-reactivating cells were detected by eGFP (green). Representative images (original magnification, 63×1.4) from 2 independent experiments are shown. (**F**) 2D10 cells were infected with HSV-2(G) (MOI = 1 PFU/cell) for 8 hours and washed, and then fresh medium or medium supplemented with PHA (10 μg/mL) or TNF (10 ng/mL) was added. The cells were fixed and stained 24 hours after HSV-2 exposure. Representative images (original magnification, 63×1.4) from 2 independent experiments are shown. (**G**) The numbers of gB^+^ (red), eGFP^+^ (green), and gB^+^/eGFP^+^ (merge) cells and total cells (blue) were quantified in 5 randomly selected fields in **E** and **F** using Cell Counter ImageJ software (NIH); pie charts show relative proportions.

To determine whether HSV-2 impacted HIV replication, CD4^+^ T cells isolated from HIV^+^ donors were activated with PHA and then infected with HSV-2(SD90) (MOI = 1) for 48 hours (*n* = 3). We used PHA rather than anti-CD3/CD28 for these studies because identifying HIV-infected cells harboring replication-competent virus in the peripheral blood is challenging and PHA has been shown to more consistently promote HIV replication (12). The cells were stained with anti-gB and anti-p24 antibodies to identify HSV-2– and HIV-1–infected cells, respectively, and analyzed by flow cytometry (Figure 3A). With the HSV-2 infection after PHA stimulation, there was an increase in the mean fluorescence intensity (MFI) of p24 staining in 3 of 3 donors compared with activation with PHA alone (*P* = 0.056, paired *t* test) (Figure 3B). In all 3 donors, the percentage of p24^+^ cells was greater in the gB^+^ than in the gB^–^ cells.

These findings suggest that HSV-2 promoted HIV-1 replication, but the frequency of HIV-1–infected primary CD4^+^ cells was (as expected) low and PHA activation was needed. To address these experimental limitations, we used the Jurkat-derived 2D10 cell line model of HIV latency (19). 2D10 cells were exposed, without prior stimulation, to live or UV-inactivated HSV-2(G) (MOI = 1) and HIV *ltr* expression measured 24 hpi. There was a 1 log_10_-fold increase in *ltr* expression in response to live, but not UV-inactivated, HSV-2(G) in comparison with uninfected cells (Figure 3C). To exclude the possibility that this response was isolate specific, we exposed 2D10 cells to 2 additional HSV-2 clinical isolates (4674 and SD90). All 3 isolates resulted in an at least 1 log_10_-fold increase in *ltr* expression in comparison with uninfected cells (Figure 3D). To validate the findings and to compare the response in HSV-2–infected and bystander cells, we conducted additional confocal microscopy studies. 2D10 cells were infected with HSV-2(G) (MOI = 1 or 10 PFU/cell) and, 24 hpi, fixed and stained for gB (conjugated to Alexa Fluor 647, red). HIV-reactivating cells were identified by expression of enhanced GFP (eGFP) (19). At MOI = 1, 13.3% of cells expressed gB and eGFP (reactivating, HSV-2–infected cells), 17.2% were GFP^+^ only (reactivating bystanders), and 7.1% were gB^+^ only (*n* = 406 cells, 5 random fields). At MOI = 10, 43.7% stained for both gB and GFP, 5.9% for GFP only, and 22.7% for gB only (*n* = 238 cells, 5 fields) (Figure 3, E and G).

To test whether HSV-2 exposure may interfere with the response to other reactivating agents, we first infected the cells with HSV-2 (MOI = 1) for 8 hours and then, after washing, treated the cells with medium alone or medium containing PHA or TNF, and analyzed the cells by confocal microscopy 24 hpi. HSV-2 itself triggered HIV reactivation in 15.24% ± 0.1% of the cells, and the response increased significantly (*P* < 0.05, 1-way ANOVA) to 41.75% ± 4.85% and 47.43% ± 5.42% when PHA or TNF, respectively, was subsequently added (mean ± SEM, *n* = 2 independent experiments) (Figure 3, F and G). The percentage of reactivating bystanders (eGFP^+^, gB^–^) increased from 10.9% ± 0.15% to 21.15% ± 0.95% (*P* < 0.01) and to 28.85% ± 2.45% (*P* < 0.05) when PHA or TNF was added to the cultures, respectively. The percentage of HIV-1^+^HSV-2^+^ dually stained cells also increased from 4.5% ± 0.25% to 20.08% ± 4.45% and 11.7% ± 7.7%, respectively. Together these results suggest that HSV-2 triggers HIV reactivation in HSV-2–infected and bystander CD4^+^ T cells in a dose-dependent manner and may act in concert with other activating stimuli.

### HSV-2 upregulates genes associated with HIV latency reversal and downregulates restriction factors.

To identify molecular mechanisms that may account for how HSV-2 promotes HIV-1 latency reversal and/or HIV-1 replication, we conducted RNA-Seq studies of HSV-2–infected CD4^+^ T cells that were isolated from leukopaks of 5 HIV-uninfected donors. The cells were activated with anti-CD3/CD28 for 72 hours and then mock-infected or infected with HSV-2(333ZAG) (MOI = 1). After 24 hours, the cells were separated into GFP^+^ and GFP^–^ populations by fluorescence-activated cell sorting (FACS). A mean of 33.82% (SD = 8.60) of cells were GFP^+^.

**Figure 4:**
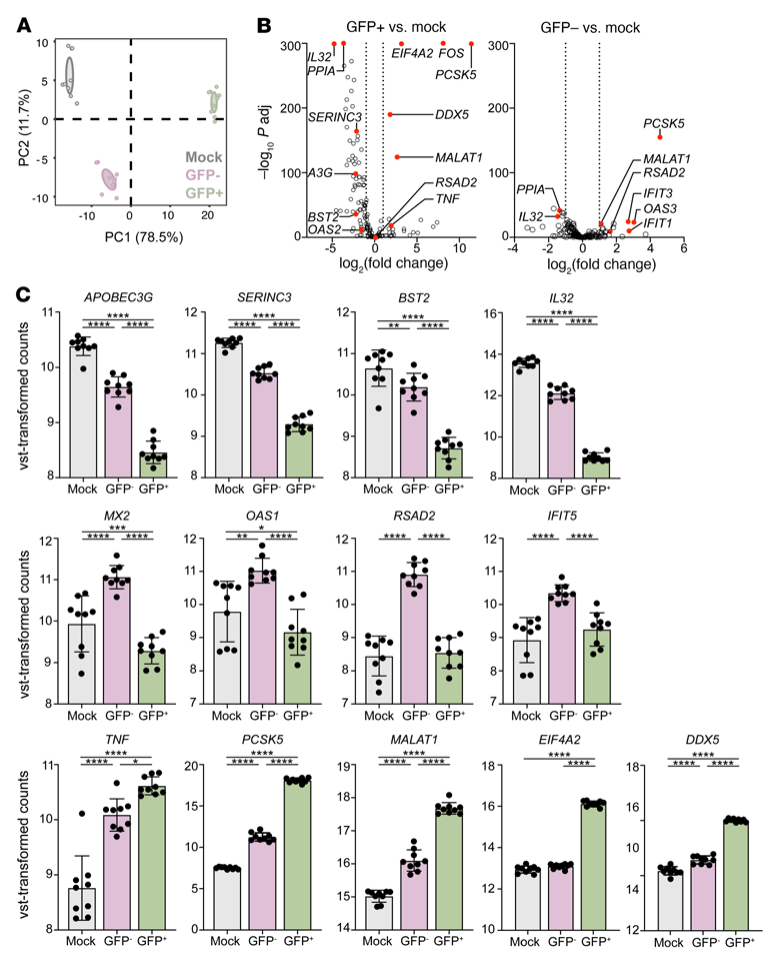
Transcriptional changes in HSV-2–infected CD4^+^ T cells. CD4^+^ T cells isolated from leukopaks of 5 healthy donors were stimulated for 72 hours by CD3/CD28 cross-linking, infected with HSV-2(ZAG) at an MOI of 10 in biological duplicate, and sorted on GFP expression, and RNA was isolated for RNA-Seq. (**A**) Principal component analysis using the genes included in GO:0009615, 0019080, and 0019058. (**B**) Volcano plots comparing expression of a subset of genes identified in our bulk RNA-Seq data selected based on their known association with HIV reactivation and replication, comparing GFP^+^ versus mock (left) and GFP^–^ versus mock (right). Select genes of interest are demarcated in red. Dotted vertical lines indicate fold change greater than 2. All demarcated genes were significant (adjusted *P* < 0.05). *A3G*, *APOBEC3G*. (**C**) Normalized count data for selected genes related to HIV infection, latency reversal, or the interferon response. *P < 0.05, **P < 0.01, ***P < 0.001, ****P < 0.0001, Wald test via DESeq2.

We identified 7,841 genes whose expression was increased significantly (log_2_ fold change > 1 and adjusted *P* < 0.01) and 6,013 whose expression was decreased significantly (log_2_ fold change > 1 and adjusted *P* < 0.01) comparing GFP^+^ and mock-infected cells. We also found significant changes in the GFP^–^ (bystander) cells with an increase in expression of 1,217 genes and a decrease in expression of 994 genes compared with mock-infected cells, although the magnitude of changes was smaller. Gene set enrichment analysis of Gene Ontology (GO) terms identified 16 ontologies with adjusted *P* values less than 0.05 that were relevant to viral processes (Supplemental Table 1). We focused on the 3 largest (GO:0009615, response to virus; GO:0019080, viral gene expression; and GO:0019058, viral life cycle), which together comprised 632 unique genes and had considerable overlap. We performed principal component analysis of these genes. The first principal component (PC) captured 78.5% of variance and differentiated the GFP^+^ samples from the GFP^–^ or mock-infected samples; PC2 captured 11.7% of variance and largely contributed to the separation of GFP^–^ and mock-infected samples (Figure 4A). The genes contributing most strongly to PC1 and PC2 are shown in Supplemental Figure 2 and Supplemental Table 2.

We compared expression of a subset of these genes, focusing on those that have been previously associated with HIV-1 latency reversal, replication, and/or pathogenesis (Figure 4, B and C, and Supplemental Table 3) (21). Among the differentially expressed genes, we found significantly increased expression in the GFP^+^ compared with mock-infected cells of the following transcripts: *PCSK5*, a proprotein convertase that promotes processing of HIV-1 gp160 (22–24); *EIF4A2*, which promotes efficient HIV-1 replication (25); *FOS*, which transcriptionally activates the HIV *ltr* promoter (26); *MALAT1*, a long noncoding RNA that may promote HIV-1 latency reversal through interactions with Polycomb repressive complex 2 (PRC2) (27); *TNF*, which promotes HIV-1 replication (28, 29); and *DDX5*, which potentiates HIV transcription (30). Conversely, we observed significantly decreased expression of *IL32*, which blocks latency reversal (12, 13); *APOBEC3G*, which inhibits HIV-1 replication (31); *SERINC3*, which reduces viral infectivity (32, 33); and *BST2* (tetherin), which inhibits release of virions from the host cell (34). *PPIA* (cyclophilin A), which has cell type–specific effects on HIV-1 (35), was also significantly downregulated in the GFP^+^ and GFP^–^ cells compared with mock-infected cells. The overall transcriptional response in the GFP^–^ cells was in a similar direction but less robust than in the GFP^+^ cells and differed with respect to several interferon-associated transcripts (*RSAD2*, *MX2*, *OAS1*, and *IFIT5*), which were significantly upregulated in the GFP^–^ compared with GFP^+^ and mock-infected cells (Figure 4C).

### Analysis of HSV-2–infected CD4^+^ T cells by single-cell RNA-Seq.

The bulk RNA-Seq analysis may fail to identify changes in subsets of T cells, and some cells that are expressed in the tissues and secondary lymph structures may be infrequently represented in the peripheral blood. Therefore, we performed single-cell RNA-Seq (scRNA-Seq) on CD4^+^ T cells isolated from human tonsil containing multiple CD4^+^ subpopulations, including T follicular helper (Tfh) cells, which our previous data have suggested are preferentially infected by HSV-2 (12). CD4^+^ T cells were isolated by negative selection, activated by anti-CD3/CD28 beads for 72 hours, and infected with HSV-2(SD90) to mirror the bulk RNA-Seq data. Gene expression was analyzed after 0 (mock), 6, and 24 hours. Cells displayed phenotypic markers of infection that increased across experimental time, including reduction in total transcript abundance (library size) (Supplemental Figure 3A) and increased expression of the HSV gene *UL15* (Supplemental Figure 3B).

**Figure 5:**
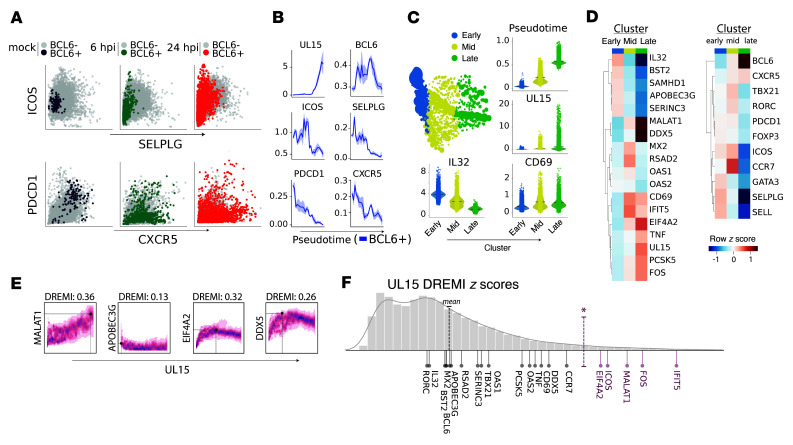
Single-cell RNA-Seq identifies features of HSV-2–infected CD4^+^ T cells. CD4^+^ T cells isolated from tonsil of an HIV^–^ donor were stimulated with anti-CD3/CD28 cross-linking and then infected with HSV-2(SD90) (MOI = 1) and subjected to single-cell RNA-Seq at 0 (mock), 6, and 24 hpi. (**A**) Scatterplots show normalized and denoised gene expression (see Methods) of cells within samples collected at different time points. Data points are colored based on upstream gating, where gray represents *BCL6^–^* cells and purple, green, and red represent *BCL6*^+^ cells from mock, 6 hpi, and 24 hpi, respectively. (**B**) Pseudotime line plots for all cells showing genes associated with infection (*UL15*) and Tfh phenotype (*BCL6*, *ICOS*, *SELPLG*, *PDCD1*, *CXCR5*). (**C**) Multiscale PHATE identifies 3 clusters of cells from all time points. Colors denote cluster identity, and the size of a dot in the embedding is proportional to the number of cells represented. Violin plots show expression of select genes organized by Multiscale PHATE cluster. Black horizontal lines represent cluster expression means, and individual points represent single cells. (**D**) Heatmaps represent expression of select genes involved in response to HSV-2 infection (left) and cell identity (right). Color scheme is based on *z* score distribution. (**E**) Mutual information (DREMI) quantified association between expression of *UL15* and select genes *MALAT1*, *APOBEC3G*, *EIF4A2*, and *DDX5* visualized with DREVI. (**F**) Histogram shows *z* score distribution of mutual information between individual genes and *UL15* for all measured transcripts, calculated with DREMI. Dashed vertical lines show mean (black) and 95% confidence (red). HSV-2 infection–related genes with *z* scores above 95% confidence (*P ≥ 0.05) are colored in red.

To confirm the presence of Tfh cells in tonsil samples, we identified a population of mock-infected *BCL6^+^* cells that were *CXCR5^hi^*, *PDCD1^hi^*, and *SELPL^lo^*, although *ICOS* was slightly lower than expected but still appreciably expressed (Figure 5A). *BCL6^+^* cells remained present following HSV-2 infection (Supplemental Figure 3C); however, the cells showed substantial dysregulation of canonical Tfh gene expression, including reduced *CXCR5* and *PDCD1* (Figure 5A). To infer gene expression dynamics across continuous infection time, we performed diffusion pseudotime analysis (36). We selected a starting point based on maximal *UL15* expression. Among the *BCL6^+^* cells, progression through pseudotime was associated with increased *UL15*, as expected based on our parameters, as well as trends toward loss in Tfh marker genes (Figure 5B), supporting the hypothesis that BCL6^+^ Tfh cells infected with HSV-2 incur disruptions in expression and loss of Tfh identity markers including reduced *CXCR5* and *PDCD1* expression.

Cell clustering and visualization were performed using Multiscale PHATE (35). We identified 3 coarse-grain clusters that captured transcriptional states associated with early, mid, and late stages of HSV-2 infection, estimated by averaged pseudotime values within clusters (Figure 5C). There was a progressive increase in *UL15* and decrease in *IL32* expression from early to late stages, and an increase in CD69 that occurred predominantly between the early and mid phases (Figure 5C). Later clusters also showed changes in genes matching the bulk RNA-Seq data, including increased *MALAT1*, *DDX5*, and *EIF4A2* and decreased *BST2*, *SAMHD1*, *APOBEC3G*, and *SERINC3* expression (Figure 5D). To identify candidate mechanisms, we quantified functional relationships between *UL15*-defined infection pseudotime and every measured gene using mutual information (conditional-Density Resampled Estimate of Mutual Information [DREMI]) visualized with conditional-Density Rescaled Visualization (DREVI) (Figure 5, E and F). Consistent with the bulk transcriptomic data (Figure 4), increased *UL15* was associated with increased *MALAT1* and *DDX5* and sharp decreases in *APOBEC3G* expression. *EIF4A2* also increased with *UL15* to a point but peaked and began to drop with higher *UL15* expression, possibly suggesting a negative regulatory mechanism or feedback loop. Consistent with pseudotime analysis, we observed dysregulation of *BCL6*, as well as loss of *RORC* and *GATA3* expression associated with progressive *UL15* expression (Supplemental Figure 4D).

**Figure 6:**
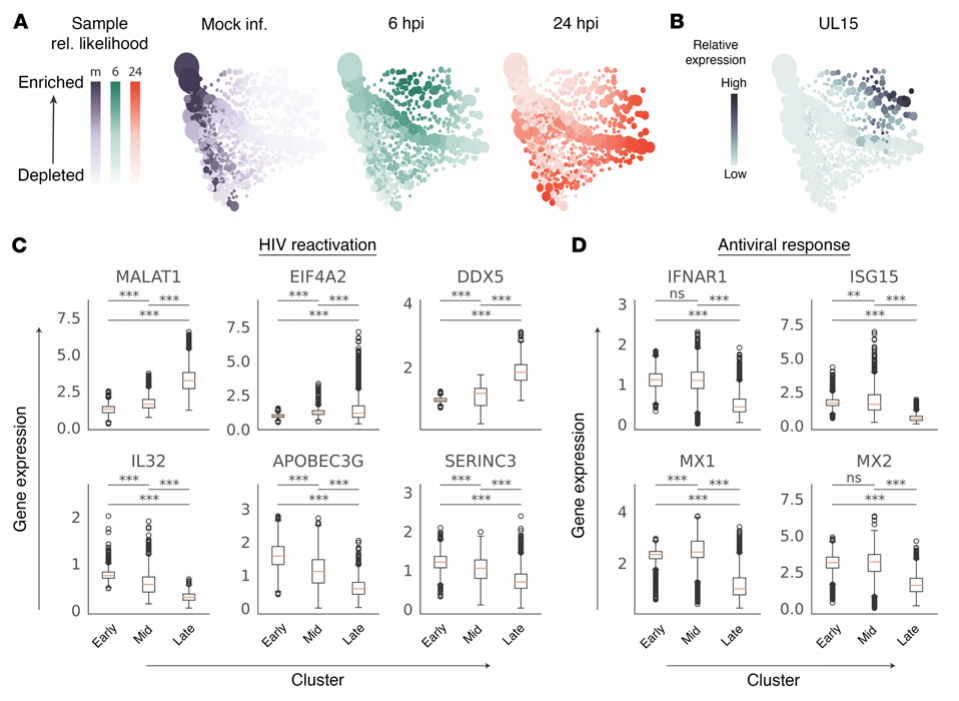
scRNA-Seq reveals shared and divergent gene expression features of HSV-2–infected and bystander cells. (**A**) Multiscale PHATE visualization of sample likelihood score calculated with MELD for each experimental time point. Purple, green, and red color schemes correspond to mock, 6 hpi, and 24 hpi, respectively, and the size of a dot is proportional to the number of cells represented. (**B**) Multiscale PHATE visualization of *UL15* expression, where color intensity denotes gene expression. (**C** and **D**) Box plots showing relative gene expression of cells at 24 hpi within Multiscale PHATE clusters for genes involved in HIV reactivation (**C**) and general antiviral immune responses (**D**). Values represent normalized and denoised gene expression levels for individual cells relative to average expression in mock-infected cells (**P < 0.01, ***P < 0.001, Welch’s *t* test). At 24 hpi, cells in the early cluster are mock-like, cells in the mid cluster are defined as bystanders, and those in the late are productively HSV-2–infected.

We next investigated transcriptional effects on “bystander cells,” which are present as a result of the relatively low MOI (MOI = 1) used in the experimental design. At each experimental time point we calculated relative sample density across the Multiscale PHATE embedding using MELD (Figure 6A) (37). In accordance with pseudotime analysis, mock-infected cells were concentrated in the early cluster and cells at 6 hpi were concentrated in the mid cluster. In contrast, cells at 24 hpi diverged and we observed populations in both mid and late clusters. Because *UL15* expression was concentrated in the late cluster (Figure 5C and Figure 6B), we consider 24 hpi cells in the mid cluster “bystander cells,” 24 hpi cells in the late cluster “infected cells,” and 24 hpi cells in the early cluster “mock-like.” We found similar trends in gene expression changes between *UL15^+^* and bystander cells for genes associated with HIV-1 reactivation such as *MALAT1*, *EIF4A2*, *DDX5*, *IL32*, *APOBEC3G*, and *SERINC3*, albeit at a lower magnitude (Figure 6C). The interferon response genes *IFNAR1*, *ISG15*, *MX1*, and *MX2* were selectively downregulated in the HSV-infected cells (Figure 6D). In total, the scRNA-Seq data suggest that within a population of CD4^+^ T cells, HSV-2 increases the global potential for HIV reactivation or replication in both HSV-2–infected (*UL15^+^*) and bystander cells.

### HSV-2 VP16 upregulates MALAT1 to promote HIV reactivation by inducing histone modifications.

**Figure 7:**
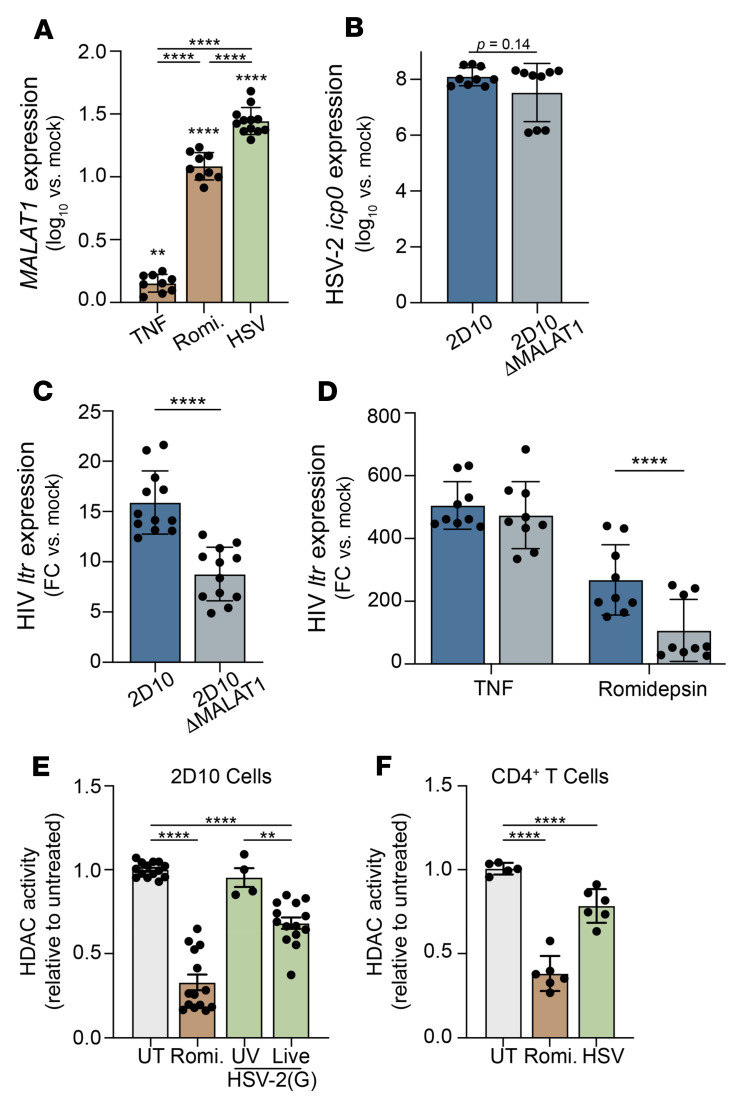
HSV-2–induced reactivation is decreased in the absence of *MALAT1*. (**A**) 2D10 cells were exposed to TNF (10 ng/mL), romidepsin (6.25 nM), or HSV-2(SD90) (MOI = 1 PFU/cell) or mock-treated, and *MALAT1* expression was determined 24 hours after treatment. Results are expressed as log_10_ fold change (FC) relative to mock-treated cells (*n* = 3 independent experiments, **P < 0.01, ****P < 0.0001, compared with mock-treated cells by 1-way ANOVA). (**B**) 2D10 or Δ*MALAT1* cells were exposed to HSV-2(SD90) (MOI = 1 PFU/cell) and HSV-2 *ICP0* expression determined 24 hpi as log_10_ fold change relative to mock-infected cells. (**C** and **D**) 2D10 or Δ*MALAT1* cells were infected with HSV-2(SD90) (MOI = 1 PFU/cell) (**C**) or treated with TNF or romidepsin (**D**), and HIV *ltr* expression was determined 24 hours after treatment. Results are presented as fold change in gene expression relative to mock-treated cells. ****P < 0.0001, unpaired *t* test comparing 2D10 vs. Δ*MALAT1* cells. (**E**) 2D10 cells were infected with live or UV-inactivated HSV-2(G) (MOI = 1), treated with romidepsin (6.25 nM), or left untreated (UT), and after 24 hours of incubation, the cells were harvested, nuclei isolated, and HDAC activity measured using a colorimetric HDAC activity assay. (**F**) Primary CD4^+^ T cells were stimulated by CD3/CD28 cross-linking for 72 hours and infected with HSV-2(G) (MOI = 1), treated with romidepsin, or left untreated (UT). HDAC activity was assayed as in **E**. **P < 0.01, ****P < 0.0001 relative to untreated cells, 1-way ANOVA.

To identify the contribution of *MALAT1* to HSV-2–mediated reactivation of latent HIV-1, we again took advantage of 2D10 cells and infected the cells with HSV-2 or treated the cells with romidepsin, a histone deacetylase inhibitor (HDACI), or TNF, which acts through the NF-κB pathway. Consistent with the response in primary CD4^+^ T cells, HSV-2 infection resulted in a 1.45 log_10_-fold increase in *MALAT1* expression relative to mock-treated cells, which was greater than the response to romidepsin (*P* < 0.0001) (Figure 7A). In contrast, TNF induced only a small increase in *MALAT1* expression.

We then deleted *MALAT1* in 2D10 cells using CRISPR/Cas9 (Δ*MALAT1*); deletion of *MALAT1* was confirmed by PCR (Supplemental Figure 4). Δ*MALAT1* cells were equally as susceptible to HSV-2 infection as parental 2D10 cells (*MALAT1^+/+^*), as assessed by quantification of *ICP0* expression by quantitative reverse transcriptase PCR (RT-qPCR) 24 hpi (Figure 7B). However, HSV-2–triggered latency reversal was reduced by about half in Δ*MALAT1* compared with *MALAT1^+/+^* cells (*P* < 0.01) (Figure 7C). Romidepsin-triggered HIV latency reversal was also reduced in the knockout compared with *MALAT1^+/+^* cells (*P* < 0.001) (Figure 7D). In contrast, there was no reduction in HIV latency reversal in Δ*MALAT1* compared with *MALAT1^+/+^* cells cultured with TNF. These findings suggest that *MALAT1* contributes to HSV-2–induced and HDACI-mediated HIV latency reversal. The mechanistic overlap between HSV-2 and pharmacological HDACI was supported by the findings that exposure of 2D10 cells to live, but not UV-inactivated, HSV-2 led to a significant reduction in HDAC activity compared with uninfected cells; similar results were observed with anti-CD3/CD28–stimulated primary CD4^+^ T cells (Figure 7, E and F).

**Figure 8:**
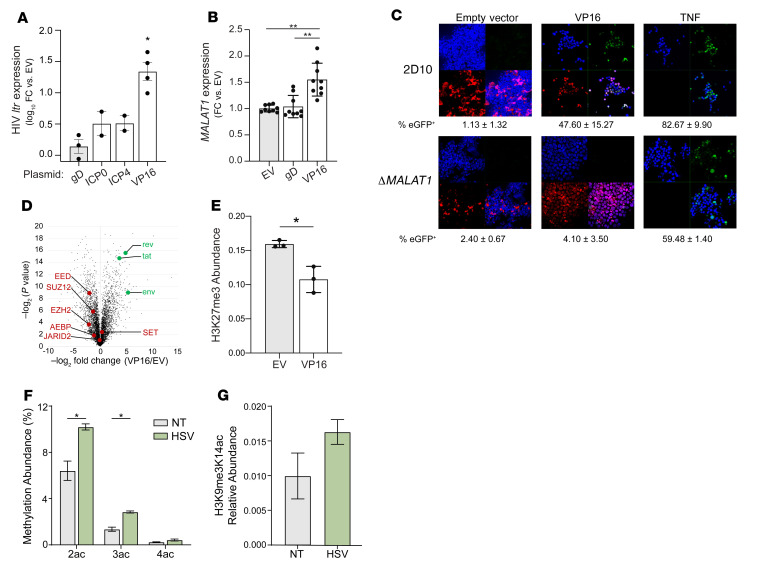
HSV-2 VP16 triggers HIV reactivation, upregulates MALAT1, and induces histone modifications. (**A**) Jurkat 2D10 cells were transfected with the indicated plasmids and mCherry-positive cells isolated 42 hours later by FACS. RNA was extracted, and log_10_ fold change (FC) in HIV *ltr* expression was quantified relative to empty vector (EV) control by RT-qPCR. (**B**) 2D10 cells were transfected with the indicated plasmids and processed as in **A**, and the fold change in *MALAT1* expression was quantified relative to EV control. (**C**) 2D10 or Δ*MALAT1* cells were transfected with VP16 or EV plasmids or treated with TNF (10 ng/mL) for 24 hours and then fixed and stained with DAPI (nuclei blue). Representative images show nuclei (blue, top left), eGFP^+^ cells (green, top right), transfected cells (red, bottom left), and merged images (bottom right) (original magnification, 63×1.4). Percentage of eGFP^+^ cells (HIV-reactivated) was quantified in 5 randomly selected fields and is indicated below. (**D** and **E**) 2D10 cells were transfected with VP16 or EV plasmids and, 42 hours later, were sorted on mCherry by FACS and analyzed for protein expression (**D**), or nuclei were extracted and histones isolated for evaluation of histone modifications (**E**). HIV proteins are highlighted in green and members of the PRC2 complex in red (**D**). (**F** and **G**) 2D10 cells were infected with HSV-2(G) at an MOI of 10 PFU/cell or mock-infected. After 24 hours of incubation, nuclei were extracted and histones isolated and evaluated by mass spectrometry. The percentages of H4 with 2, 3, or 4 acetylations (**F**) and acetylation of histone H3 already modified with the silencing modification H3K9me3 (**G**) were quantified. *P < 0.05, **P < 0.01, 1-way ANOVA (**A** and **B**) or unpaired *t* test (**E**–**G**).

To identify which HSV-2 genes upregulate *MALAT1* to promote HIV-1 latency reversal, 2D10 cells were transfected with plasmids coding for VP16, ICP0, or ICP4, three HSV proteins associated with histone modifications and/or recruitment of transcription factors to viral gene promoters (38, 39). Plasmids encoding HSV-2 glycoprotein D (gD), which is involved in viral entry but does not regulate transcription, and expressing only cherry red (empty vector [EV]) were included as controls. Transfected cells were identified and sorted by cherry red expression (Supplemental Figure 5). Transfection with VP16 induced the greatest increase in HIV-1 *ltr* (1.34 log_10_-fold increase, *P* = 0.016) (Figure 8A) and *MALAT1* expression (1.55-fold, *P* = 0.0052) relative to EV-transfected cells (Figure 8B). ICP0 and ICP4 had more modest effects on HIV-1 *ltr* expression. Transfection of the *MALAT1^+/+^* cells with VP16 caused increased HIV reactivation, visualized by confocal microscopy (Figure 8C). However, the response to VP16 was abrogated in the *MALAT1* cells. There was no significant difference in the response to TNF in *MALAT1^+/+^* versus Δ*MALAT1* cells.

Proteomic and histone analysis of 2D10 cells transfected with VP16 or EV showed a decrease in abundance of proteins constituting PRC2, which mediates addition of the trimethylation of lysine 27 on histone 3 protein (H3K27me3) silencing mark (Figure 8D and Supplemental Table 4). Furthermore, analysis of nuclear histones showed that the global H3K27me3 abundance was reduced in VP16-transfected cells compared with EV control (*P* < 0.05) (Figure 8E and Supplemental Table 5). Histone analysis of HSV-2–infected 2D10 cells (MOI = 10) showed a significant increase in global levels of histone H4 acetylation, which benchmarks an overall decondensation of the host chromatin (Figure 8F). In addition, there was increased acetylation on histone H3 already modified with the silencing modification H3K9me3 (Figure 8G and Supplemental Table 6). The extensive expression of the HSV-2 proteome (Supplemental Figure 6 and Supplemental Table 7) obscured some of the regulations we observed in our simpler model of VP16 transfection. For instance, we still observed a downregulation of the PRC2 complex, but not all proteins of the complex passed the significance threshold.

## Discussion

The studies presented here confirm the susceptibility of activated human CD4^+^ T cells to HSV-2 and uncover transcriptional changes elicited by HSV-2 that may promote HIV-1 latency reversal and replication and thus contribute to the HIV/HSV-2 syndemic. HSV-2 infection led to downregulation of HIV-1 restriction factors such as *APOBEC3G* and *IL32*, and increased expression of *MALAT1*, *DDX5*, and other genes that potentially drive HIV replication. The transcriptional responses were of a greater magnitude in HSV-2–infected cells but were also observed in bystanders.

The importance of *MALAT1* in driving HSV-induced reversal of HIV-1 latency is supported by our bulk and single-cell RNA-Seq data including the DREMI analysis with primary CD4^+^ T cells and the finding that CRISPR/Cas9–mediated deletion of *MALAT1* in 2D10 cells resulted in a reduction in HSV-2–mediated HIV reactivation. A similar reduction in latency reversal was observed when the knockout cells were treated with romidepsin but not when they were treated with TNF, which reactivates HIV-1 by mechanisms involving activation of NF-κB (40, 41). We mapped the HSV-induced upregulation of *MALAT1* and decrease in global H3K27me3 abundance to the HSV tegument protein VP16, an important transactivator that also triggers HSV reactivation in neuronal cells by modification of repressive histone marks and upregulation of lytic gene transcripts (42). However, the epigenetic response to HSV-2 is more complex than VP16-mediated upregulation of *MALAT1*, as infection led to other epigenetic changes including an increase in histone acetylations, including histones already modified with silencing marks such as H3K9me3. The increase in histone acetylations is consistent with studies of HSV-1 lytic infection of epithelial cells (43).

*MALAT1* is a long intergenic noncoding RNA that is highly expressed in the nucleus of cells. It regulates transcription by interacting with transcription factors to activate or repress their activity through epigenetic modulation in several disease processes, including cancer. It is one of the most highly expressed transcripts in naive CD4^+^ T cells and has been shown to be downregulated in response to T cell activation and other infections (44, 45). Thus, the observation that HSV-2 significantly increased the expression of *MALAT1* in CD4^+^ T cells was unanticipated. The impact of *MALAT1* on the immune response to infections is complex and may be pathogen specific. For example, *MALAT1* knockdown in mice was associated with enhanced clearance of visceral leishmaniasis but more severe disease in a model of malaria (44). Possibly the upregulation of *MALAT1* by HSV-2 promotes viral replication as the lncRNA regulates cell cycle progression (46).

Two prior studies have suggested a link between *MALAT1* and HIV-1 replication. *MALAT1* transcript levels were higher in PBMCs isolated from HIV-infected, antiretroviral-naive patients and were reduced following treatment (47). *MALAT1* was shown in vitro to interact with EZH2, the core catalytic component of PRC2, which resulted in decreased H3K27me3 to relieve epigenetic silencing of HIV transcription (27). It has been suggested that the *MALAT1*-PRC2 interaction alters the target specificity of PRC2, directing it away from the *ltr* promoter and to other genomic loci (27, 47). Our findings suggest that HSV VP16 promotes HIV-1 latency reversal primarily through this mechanism as evidenced by the increase in *MALAT1* expression, decrease in global H3K27me3 abundance in VP16-transfected 2D10 cells, and loss of VP16-mediated HIV latency reversal in *MALAT1*-knockout cells. However, we did not specifically measure histone methylations of the HIV *ltr*. Notably, the genomic locus for *MALAT1*, chromosome 11q13, is a known hotspot for HIV integration into the host genome (48). This may contribute to its effects on the HIV-1 *ltr*, since *MALAT1* has a preferential regulatory effect on neighboring genes (49), although in 2D10 cells, the provirus is integrated in chromosome 16 (19).

While the DREMI analysis demonstrated a strong temporal association of *MALAT1* and HSV *UL15*, persistently high or increased expression of *MALAT1* was not restricted to the *UL15^+^* cluster in the pseudotime analysis. However, the *UL15^+^* cluster also expressed higher levels of other transcripts associated with HIV transcription, including *EIF4A2* and *DDX5*, and, conversely, lower levels of HIV restriction factors such as *APOBEC3G*, which were also strongly associated with *UL15* in the DREMI analysis. Although we observed shared gene expression trends between cells identified as “bystanders” (24 hpi cells in the *UL15^–^* mid cluster) and “infected” (24 hpi cells in the *UL15^+^* late cluster), the magnitude of change was generally lower. Thus, HSV may promote latency reversal in a manner dependent on coexpression of *MALAT1* and other genes, and the partial transcriptional response in uninfected bystander cells may potentiate HIV reactivation.

**Figure 9:**
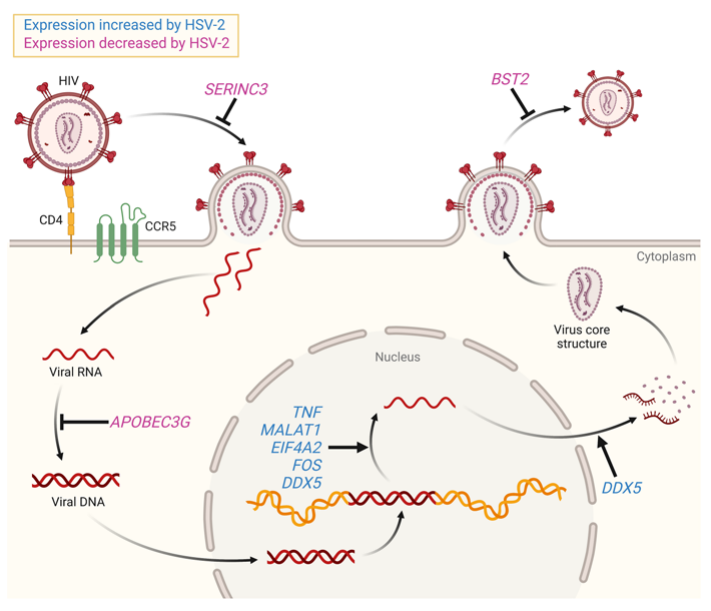
HSV-2–associated transcriptional changes promote HIV infection, replication, and virion production. Shown are selected transcriptional changes identified in bulk and/or single-cell RNA-Seq analyses and their effects on HIV infection, replication, or virion production. Relationships shown with an arrow promote the indicated process; relationships shown with a blunted arrow inhibit the indicated process. Genes whose expression was increased in HSV-2–infected cells are shown in blue; those whose expression was decreased are shown in purple.

*MALAT1* knockdown abrogated the response to VP16 but only reduced HIV latency reversal by approximately 50% in response to HSV-2 infection, indicating the contribution of *MALAT1*-independent pathways in promoting HIV latency reversal. In addition to increased histone acetylations in response to HSV-2 infection, which would lead to decondensation of chromatin and drive HIV gene expression, we observed other transcriptional changes in the bulk and scRNA-Seq studies that could act on different steps to promote HIV-1 reactivation and replication, as illustrated in Figure 9. HSV decreased *SERINC3*, which blocks viral entry (33); *APOBEC3G*, which inhibits viral transcription by generating G-to-A mutations; *BST2* (tetherin), which prevents viral particle release (34); and *IL32*, which we had previously identified and may block HIV-1 transcription (12, 50). Conversely, HSV-2 increased *EIF4A2* and *TNF*, which promote HIV-1 replication (25, 28, 29); *PCSK5*, which promotes processing of HIV gp160 (22–24); *FOS*, which may transcriptionally activate the HIV-1 *ltr* promoter (26); and *DDX5*, which potentiates HIV-1 transcription as a cofactor for tat (30, 51).

In peripheral blood CD4^+^ T cells, HSV infection was greatest in memory T cells and specifically in cells that express transcription factors associated with Th1, Th17, and Tfh identity. Although we cannot distinguish CD45RA/CD45RO isoforms in our data, we identified a population of tonsillar *BCL6^+^PDCD^hi^CXCR5^hi^SELPLG^lo^* cells that phenotypically resemble Tfh cells except for the modest expression of *ICOS*. However, by modeling continuous expression dynamics using pseudotemporal ordering, we found that these features are generally lost during the progression of HSV infection, which is further supported by DREMI analysis showing dysregulation of T cell identity transcription factor expression.

There are several limitations to these in vitro studies. First, the RNA-Seq analyses were conducted with anti-CD3/CD28–activated CD4^+^ cells from HIV^–^ donors (peripheral blood for bulk and tonsillar for single-cell). HIV infection may affect the transcriptome and thus modify the response to HSV-2. Second, while several studies have shown that HSV-2 coinfection is associated with an increase and persistence of activated CD4^+^ T cells at sites of HSV-2 reactivation (local) as well as in the peripheral blood (5–7, 12), the mechanism by which the cells are activated in vivo may differ from in vitro activation with anti-CD3/CD28 and thus impact the transcriptional response. Third, the net effect of HSV-2 in situ is likely to be more complex and to be modified by direct or indirect effects of the virus (e.g., release of cytokines, chemokines, and antimicrobial peptides) on other cell types, including epithelial cells, the primary target of HSV-2 replication, dendritic cells, and macrophages. Prior studies have shown that HSV may enhance HIV reactivation indirectly via mucosal epithelial cells through release of soluble mediators such as TNF (52), and the latter immune cell populations are susceptible to HIV-1 and HSV infection and harbor HIV reservoirs (53, 54). Similarly, there may be effects of HSV-2 on cytolytic CD8^+^ T cells that control HIV replication.

We recognize that the likelihood that HSV-2 will infect any given HIV-infected (latent or replicating) activated CD4^+^ T cell is low. However, HSV-2 reactivates often, particularly in HIV^+^ individuals (11, 55, 56). This, combined with the transcriptional changes identified in both the HSV-2–infected and bystander CD4^+^ T cells, supports a perpetuating cycle in which HSV-2 transcriptionally modifies CD4^+^ T cells, rendering them vulnerable to new HIV infection, reactivation, and replication.

In summary, we demonstrated that HSV-2 impacts the transcriptional profile of CD4^+^ T cells, including upregulation of *MALAT1*. These changes have the potential to drive HIV replication and reactivation in HIV-infected cells and to render HIV-uninfected cells more susceptible to new infection, and may result in slower HIV reservoir decay (57). These interactions not only fuel the HIV-1 epidemic but render eradication efforts more difficult to achieve. Defining mechanistically how HSV-2 coinfection contributes to HIV-1 replication and the maintenance and expansion of HIV-1 reservoirs will facilitate the identification of new strategies to disrupt the HIV-1/HSV-2 syndemic.

## Methods

### Cells and viruses.

Vero cells (monkey kidney epithelial cell line; ATCC CCL-81) and HaCaT cells (human keratinocytes; CLS Cell Lines Service 300493) were maintained in DMEM (11965-092, Thermo Fisher Scientific) supplemented with 10% (vol/vol) FBS (SH30910.03, HyClone) and penicillin/streptomycin (15140163, Thermo Fisher Scientific). Jurkat and 2D10 cells were maintained in RPMI medium (11875-093, Thermo Fisher Scientific) supplemented as for DMEM. PBMCs from HIV^–^ donors were obtained from the New York Blood Bank, and from HIV^+^ donors from a biorepository at Rockefeller University (New York, New York, USA). CD4^+^ T cells were isolated by negative selection with an EasySep Human CD4^+^ T Cell Enrichment Kit (19052, STEMCELL Technologies) and cultured in RPMI medium with 50 U/mL recombinant human IL-2 (R&D Systems). Primary CD4^+^ T cells were activated for 72 hours with 25 μL/mL ImmunoCult Human CD3/CD28 T Cell Activator (10971, STEMCELL Technologies). HSV-2(G) (58), HSV-2(333ZAG) (20), and clinical isolates 4674 (59) and SD90 (60) were propagated on Vero cells. Virus was inactivated by UV exposure for 30 minutes 10 cm from the light source.

### HSV-2 infection assays.

Cells were infected in suspension (T cells) or as adherent monolayers (HaCaT and Vero) with HSV-2 at indicated MOI (based on Vero cell titer) at 37°C for 2 hours, washed twice, and resuspended or overlaid with fresh medium. Multistep HSV growth kinetics were assessed by infection of CD4^+^ T cells and HaCaT cells with SD90 at 0.001 PFU/cell. Culture supernatants were harvested at indicated times, centrifuged at 500*g* for 5 minutes at 4°C, and frozen at –80°C. Viral titer was determined by plaque assay on Vero cells. Cell viability was determined by vital dye exclusion using a Countess II cell counter (Invitrogen).

### RNA isolation and quantitative real-time PCR.

RNA was extracted from cells with RNeasy Plus Mini kit (74134, Qiagen) or, if fewer than 10^5^ cells, with miRNeasy Micro Kit (217084, Qiagen). One hundred nanograms of RNA was used for cDNA synthesis using the High-Capacity cDNA Reverse Transcription Kit with RNase inhibitor (4374966, Thermo Fisher Scientific). Primer/probes for qPCR were: CD69-FAM (Hs00934033_m1; Thermo Fisher Scientific), IL-32–FAM (Hs00992441_m1; Thermo Fisher Scientific), MALAT1-FAM (Hs00273907_s1; Thermo Fisher Scientific), ICP0-FAM (forward: 5′-GGTCACGCCCACTATCAGGTA-3′; reverse: 5′-CCTGCACCCCTTCTGCAT-3′; probe: 5′-FAM-CAACGGAATCCAGGTCTTCATGCACG-TAMRA-3′), HIV-1 *ltr* (forward: 5′-CACACAAGGCTACTTCCCTGA-3′; reverse: 5′-TCTCTGGCTCAACTGGTACTAGCTT-3′; probe: 5′-FAM-AGAACTACACACCAGGGCCAGGGATCAG-TAMRA-3′), and RPLP0-VIC (4326314E; Thermo Fisher Scientific). Targets were amplified in 10 μL reactions in a QuantStudio 7 Flex Real-Time PCR System (Thermo Fisher Scientific); data were analyzed using QuantStudio software. Quantification was normalized against the housekeeping gene *RPLP0* in the same RNA extracts; relative gene expression was calculated using the 2^–ΔΔCt^ method.

### Flow cytometry.

Cells were harvested and washed twice in PBS (21-040-CV, Corning) and stained with Zombie NIR (423106, BioLegend) for 15 minutes at room temperature. Cells were washed 3 times in 200 mL of FACS buffer (PBS without Ca^2+^ or Mg^2+^ supplemented with 10% FBS) and stained with fluorophore-conjugated antibodies prepared in a mixture of FACS buffer and Brilliant Stain Buffer (566349, BD Biosciences) for 30 minutes at 4°C. Samples were washed 3 times, fixed in 2% paraformaldehyde for 20 minutes at room temperature, and washed 3 times before analysis. For transcription factor or p24 staining, surface-stained and fixed cells were permeabilized in 0.1% Triton X-100 for 5 minutes at room temperature. Antibodies are shown in Supplemental Table 8. Samples were acquired on a Cytek Aurora flow cytometer (Cytek Biosciences) and analyzed with FlowJo (FlowJo LLC).

### Confocal microscopy.

Cells were grown on glass coverslips, transfected with VP16 or empty vector plasmids for 48 hours, and infected or treated with HSV-2(G), TNF (10 ng/mL), or PHA (10 μg/mL) for indicated times. Cells were fixed with 4% paraformaldehyde (Electron Microscopy Sciences) and stained with DAPI (D1306, Invitrogen) to identify nuclei and with Alexa Fluor 647–conjugated anti–HSV gB (red) after permeabilization with 0.1% Triton X-100 for 3 minutes. Images were acquired by a Leica SP8 laser confocal microscope equipped with oil immersion objectives 63×1.4. Images were captured using 405 nm excitation lines, with a white-light laser range of 470–670 nm for Alexa Fluor 647, eGFP, and cherry red, and collected by adjustable emission windows. All images were captured using 2 single-molecule detection HyDs (Leica Microsystems) and 1 photomultiplier tube and processed by LAS X software (Leica Microsystems). The numbers of nuclei and GFP-positive (HIV^+^), cherry red–positive (transfected), and Alexa Fluor 647–positive (gB^+^) cells were quantified using Cell Counter ImageJ software (NIH) in 5 randomly selected fields and percentages quantified.

### Isolation of CD4^+^ T cells from human palatine tonsil.

Human palatine tonsillar tissue was obtained from a patient undergoing elective tonsillectomy, placed into 30 mL RPMI 1640 supplemented with 10% (vol/vol) FBS, 100 U/mL penicillin, 100 μg/mL streptomycin, 0.25 μg/mL amphotericin B (15240062, Gibco), and 5 μg/mL gentamicin (17-518L, Lonza) (RPMI-t), and processed within 2 hours. Tissue was cut and dissociated into single-cell suspensions by passing through a 70 μm mesh strainer, pelleted, and washed twice in cold RPMI-t. Cells were resuspended in RPMI-t, overlaid on Ficoll, and centrifuged at 1,200*g* for 20 minutes. Cells isolated from the interphase were washed twice with RPMI-t and CD4^+^ T cells isolated by negative selection.

### Bulk RNA-Seq.

CD4^+^ T cells from 5 healthy donor leukopaks (New York Blood Center) were stimulated with ImmunoCult Human CD3/CD28 T Cell Activator (0971, STEMCELL Technologies) for 72 hours, mock-infected, or infected with HSV-2(333ZAG) at MOI = 10. Cells were sorted on GFP by FACS at 24 hpi, and RNA was isolated (miRNeasy Micro Kit, 217084, Qiagen). Libraries were prepared at the Yale Center for Genome Analysis. mRNA was purified from 200 ng of RNA with oligo-dT beads (mRNA Hyper Prep, catalog KR1352, Roche KAPA Biosystems). Following first-strand synthesis with random primers, second-strand synthesis and A-tailing were performed with dUTP for generation of strand-specific sequencing libraries. Adaptor ligation with 3′-dTMP overhangs was ligated to library insert fragments. Library amplification amplifies fragments carrying the appropriate adaptor sequences at both ends. Indexed libraries were quantified by RT-qPCR (KK4854, Roche KAPA Biosystems). Insert size distribution was determined using an Agilent Bioanalyzer. Samples with a yield of ≥0.5 ng/μL and a size distribution of 150–300 bp were used for sequencing.

Individual samples were normalized to 1.2 nM, loaded on an Illumina NovaSeq flow cell, and sequenced on an Illumina NovaSeq 6000 using 100 bp paired-end reads to generate 30 million read pairs per sample. Samples were checked for read quality and adaptor contamination using FastQC and aligned to transcripts using the GENCODE transcript sequences (v33) as the reference file with Salmon (61).

Analyses were performed using R version 4.0.3. Transcripts were mapped to genes using tximport. Differential gene expression analysis was performed with DESeq2 (62). Heatmaps were generated using the ComplexHeatmap package. Principal component analysis (PCA) was performed with the prcomp function using all genes with a nonzero total read count. Before PCA, data were transformed with the vst function in DESeq2. PCA results were visualized with the factoextra package. For gene set enrichment analyses, GO (c5.go) data sets were downloaded from MSigDB (Broad Institute) and analysis performed in R with the fgsea package using 1,000 permutations.

### Single-cell RNA-Seq.

Tonsillar CD4^+^ T cells were stimulated with anti-CD3/CD28 and infected with HSV-2(SD90) (MOI = 1). Cells were harvested at 0 (mock), 6, and 24 hpi, and dead cells were depleted by FACS. Single-cell libraries were prepared at the Yale Center for Genome Analysis. Gel/bead emulsions were prepared by loading of single-cell suspensions in RT Master Mix (Thermo Fisher Scientific) on the Single Cell A Chip (10x Genomics) with a pool of 750,000 barcoded gel beads each containing (a) an Illumina R1 sequence (read 1 sequencing primer), (b) a 16 nt 10× barcode, (c) a 10 nt unique molecular identifier (UMI), and (d) a poly-dT primer sequence. Leftover biochemical reagents and primers were removed using silane magnetic beads. Full-length, barcoded cDNA was then amplified by PCR to generate sufficient mass for library construction. Enzymatic fragmentation and size selection were used to optimize the cDNA amplicon size prior to library construction. R1 (read 1 primer sequence) was added during GEM incubation. P5, P7, a sample index, and R2 (read 2 primer sequence) were added during library construction via end repair, A-tailing, adaptor ligation, and PCR. The final libraries contain the P5 and P7 primers used in Illumina bridge amplification. Demultiplexing, alignment, and gene counting were performed in Cell Ranger (10x Genomics). Sequencing reads were aligned to hg38 and the HSV-2(SD90) genome reference (63).

Cells with a library size of fewer than 1,000 or more than 40,000 UMIs were removed. Genes observed in fewer than 10 cells were removed. To filter dead or dying cells while accounting for the increase in mitochondrial gene expression following HSV infection, cells expressing greater than 60% mitochondrial genes were removed. Mitochondrial and ribosomal genes were excluded from downstream analysis. The filtered gene expression matrix was library size normalized and square root transformed. Before individual gene analysis, MAGIC was used to denoise data and account for technical dropout with hyperparameters knn = 3 and decay = 3 (64).

For analysis of Tfh-associated genes, an expression cutoff of 0.25 was set to discriminate *BCL6^+^* and *BCL6^–^* based on global distribution of gene expression values. Pseudotemporal ordering was performed with diffusion pseudotime (36) using the cell with the highest *UL15* as the root. The reverse trajectory was calculated by subtraction of 1– (pseudotime vector) to set the directionality of ordering toward infection.

Multiscale PHATE was used with default parameters for cell clustering and visualization using scaled normalized gene expression values as the input (65). Multiscale PHATE combines PHATE (potential of heat-diffusion for affinity-based trajectory embedding), a manifold-preserving dimensionality reduction method that preserves local and global distances, with iterative coarse-graining of data points called diffusion condensation, to produce robust embeddings and clusters of data at multiple scales of resolution. Diffusion condensation scales for clusters were selected based on biological interpretation that coarse-grain clusters captured progressive stages of infection, supported by downstream cluster analysis.

Conditional-Density Resampled Estimate of Mutual Information (DREMI) and conditional-Density Rescaled Visualization (DREVI) were used to quantify and visualize association between UL15 and all detected genes (64). DREMI predicts relationships by estimating how effectively the expression of gene X can be used to predict the expression of gene Y. DREMI is shape agnostic and accounts for non-uniform density, making it a useful metric for describing gene expression relationships that do not consistently occur in regular patterns (e.g., linear correlation) and for which a large amount of information may be contained in rare cell states. Genes were selected for downstream analysis based on a 95% confidence level.

Sample density was estimated using MELD (37). For each time point, we considered a set of binary labels on all cells where 1 denotes that the cell originally came from that sample, and 0 denotes that it came from a different sample. We considered these discrete signals on the data graph, which was inputted to MELD to estimate continuous density at each data point, and normalized to calculate a likelihood score for each cell. For Multiscale PHATE, cell likelihood scores were mapped to condensed data points.

### Plasmid construction and transfection.

VP16 or glycoprotein D (gD) was amplified from cDNA generated from HSV-2(G)–infected 2D10 cells and cloned into the NheI and BamHI (for VP16) or AgeI (for gD) sites of the mCherry2 N1 plasmid (54517, Addgene). To construct ICP0-mCherry and ICP4-mCherry, mCherry2, obtained from AgeI and EcoRI digestion of mCherry C1 plasmid (54563, Addgene), was cloned into AgeI and EcoRI sites of ICP0-eGFP (66) and ICP4-eYFP (67) to replace the eGFP and eYFP, respectively. 2D10 cells were electroporated with plasmid DNA using Cell Line Nucleofector Kit V (VCA-1003, Lonza) and program C-016. After electroporation, cells were transferred to a 6-well plate and incubated at 37°C, 5% CO_2_, for 42 hours until FACS. For Lipofectamine-based transfections, transfection complexes were prepared using Lipofectamine 2000 (Thermo Fisher Scientific).

### CRISPR/Cas9–mediated deletion of MALAT1.

CRISPR/Cas9 knockouts of *MALAT1* in 2D10 (68) cells were produced using the pSpCas9(BB)-2A-Puro (PX459) V2.0 plasmid, a gift from Feng Zhang (plasmid 62988, Addgene). Plasmids were modified by insertion of 20 bp guide DNA segments (three 5′ and three 3′ guide targets) selected by scoring on CRISPick (Broad Institute; https://portals.broadinstitute.org/gppx/crispick/public) and CRISPOR (http://crispor.tefor.net/). Sequence-verified plasmids were mixed with cells in individual sets of 5′ and 3′ guide pairs. 2D10 cells in exponential growth phase were suspended in modified Amaxa buffer (69), mixed with 15 μg each of paired 5′ and 3′ guide/Cas9 plasmids on ice, and electroporated at 250 V and 960 μF using a Bio-Rad GenePulser XCell exponential decay waveform. After electroporation, cells were cultured for 2 days before passaging and selection with one 4-day passage of 0.40 μg/mL puromycin (58-58-2, R&D Systems) followed by 3 days of 0.25 μg/mL puromycin (69). Surviving cells were cloned at limiting dilution, and genomic DNA was prepared with a DNeasy Blood and Tissue kit (Qiagen) and screened by PCR for knockout-specific (across the repair junction) and wild-type (within the 10,727 bp segment targeted for deletion) bands. The effective guides were 5′-TTAGTGACGCTGTATAGGTT at the 5′ end and 5′-GGCCAAGGCCTATACAGGCT at the 3′ end (both on the reverse strand); knockout primers were F-TCCCCAACACCGTACACAACCTG and R-GAGCACCTAGCTCAGCTTCTTTCA (209 bp product), and wild-type primers were F-CCCCTCACCTCGATGCAGCCAGTA and R-AAGACGCCGCAGGGATTTGAACC (577 bp product) (Supplemental Figure 3).

### HDAC activity assay.

HSV-2–infected or romidepsin-treated cells were fractionated with Minute Cytoplasmic & Nuclear Extraction Kits (SC-003, Invent Biotechnologies Inc.), and at least 0.5 μg of nuclear extract was used to determine HDAC activity (Epigenase HDAC Activity/Inhibition Direct Assay Kit, P-4034-96, EpigenTek). Absorbance was measured using a SpectraMax M5e microplate reader and SoftMax Pro 7.1 GxP software (Molecular Devices).

### Proteomic and histone preparation and analyses.

Cells were harvested in PBS, pelleted, resuspended, divided into 2 aliquots, flash-frozen in liquid nitrogen, and stored at –80°C. One aliquot was used for isolation of peptides for proteome studies and the other for histone analysis (70); details are provided in Supplemental Methods. Proteome raw files were searched with Proteome Discoverer software (v2.4, Thermo Fisher Scientific) using SEQUEST search engine and the SwissProt human and HIV database. The search for total proteome included variable modification of N-terminal acetylation and fixed modification of carbamidomethyl cysteine. Trypsin was specified as the digestive enzyme with up to 2 missed cleavages allowed. Mass tolerance was set to 10 ppm (for precursor ions and 0.2 Da for product ions. Peptide and protein false discovery rate was set to 1%. Proteins were log_2_-transformed and normalized by the average value of each sample, and missing values were imputed using a normal distribution 2 standard deviations lower than the mean (71). Statistical regulation was assessed using a heteroscedastic *t* test (if *P* value was less than 0.05). Data distribution was assumed to be normal.

Histone peptide raw files were imported into EpiProfile 2.0 software (72). From the extracted ion chromatogram, the area under the curve was obtained and used to estimate the abundance of each peptide. To achieve the relative abundance of posttranslational modifications, the sum of all different modified forms of a histone peptide was considered as 100%, and the area of the particular peptide was divided by the total area for that histone peptide in all of its modified forms. The relative ratio of 2 isobaric forms was estimated by averaging of the ratio for each fragment ion with different mass between the 2 species. The resulting peptide lists generated by EpiProfile were exported to Microsoft Excel for analysis.

### Statistics.

RNA-Seq data were deposited in the NCBI’s Gene Expression Omnibus database (GEO GSE229392; GSE229390 [bulk] and GSE229391 [single-cell]). Data analyses were performed using GraphPad Prism version 9.4.0 software (GraphPad Software Inc.). Statistical analyses of RT-qPCR data were performed using log_10_-transformed values including where data are presented as non-transformed values. Data were tested for normality and analyzed using parametric (normally distributed) or non-parametric (non-normally distributed) tests; *t* tests were 2-tailed. *P* values less than 0.05 were considered significant.

### Study approval.

Collection of human CD4^+^ T cells and tonsillar tissue was approved by the Albert Einstein College of Medicine IRB (2015-5463 and 2019-10344, respectively).

## Author contributions

CAP, LNL, KCH, and BCH designed the study. CAP, LNL, PPH, NC, LK, FZ, and S Stransky performed experiments and data analysis. HRS, S Sidoli, WP, and SK performed data analysis. MN provided tonsillar tissue. CAP, HRS, KCH, and BCH wrote the manuscript, and all authors edited the manuscript.

## Supplementary Material

Supplemental data

Supplemental table 4

Supplemental table 5

Supplemental table 6

Supplemental table 7

## Version 1 04/20/2023

In-Press Preview

## Version 2 06/01/2023

Electronic publication
