# Supplementary material for: HSV-2 triggers upregulation of MALAT1 in CD4+ T cells and promotes HIV latency reversal
Source: J Clin Invest. 2023 Jun 1;133(11):e164317. doi: 10.1172/JCI164317 (PMC10232005; doi:10.1172/JCI164317)

Supplemental materials

Supplemental Methods:

Preparation of samples for proteome and histone analysis: Cells were thawed on ice and lysed for 1 h in buffer containing 5% SDS, 5 mM DTT and 50 mM ammonium bicarbonate (pH = 8), alkylated with 20 mM iodoacetamide in the dark for 30 minutes, and then phosphoric acid was added at a final concentration of 1.2%. Samples were diluted in six volumes of binding buffer (90% methanol and 10 mM ammonium bicarbonate, pH 8.0). After gentle mixing, the protein solution was loaded to an S-trap filter (Protifi) and spun at 500 g for 30 sec. The sample was washed twice with binding buffer. 1 µg of sequencing grade trypsin (Promega), diluted in 50 mM ammonium bicarbonate, was added into the S-trap filter and samples were digested at 37°C for 18 h. Peptides were eluted in three steps: (i) 40 µl of 50 mM ammonium bicarbonate, (ii) 40 µl of 0.1% TFA and (iii) 40 µl of 60% acetonitrile and 0.1% TFA. The peptide solution was pooled, spun at 1,000 g for 30 sec and dried in a vacuum centrifuge.

Histones were acid-extracted with chilled 0.2 M sulfuric acid (5:1, sulfuric acid: pellet) and incubated with constant rotation for 4 h at 4°C, followed by precipitation with 33% trichloroacetic acid (TCA) overnight at 4°C (69). The supernatant was removed, tubes rinsed with ice-cold acetone containing 0.1% HCl, centrifuged and rinsed again using 100% ice-cold acetone. After the final centrifugation, the supernatant was discarded, and the pellet was dried using a vacuum centrifuge. The pellet was dissolved in 50 mM ammonium bicarbonate, pH 8.0, and histones were subjected to derivatization using 5 µL of propionic anhydride and 14 µL of ammonium hydroxide (all Sigma Aldrich) to balance the pH at 8.0. The mixture was incubated for 15 min and the procedure was repeated. Histones were then digested with 1 µg of sequencing grade trypsin (Promega) diluted in 50mM ammonium bicarbonate (1:20, enzyme:sample) overnight at RT. Derivatization reaction was repeated to derivatize peptide N-termini. The samples were dried in a vacuum centrifuge. Prior to mass spectrometry analysis, samples were desalted using a 96-well plate filter (Orochem) packed with 1 mg of Oasis HLB C-18 resin (Waters). Samples were resuspended in 100 µl of 0.1% TFA and loaded onto the HLB resin, which was preequilibrated

using 100 µl of the same buffer. After washing with 100 µl of 0.1% TFA, samples were eluted with a buffer containing 70 µl of 60% acetonitrile and 0.1% TFA and dried in a vacuum centrifuge.

Proteome and histone samples were resuspended in 10 µl of 0.1% TFA and loaded onto a Dionex RSLC Ultimate 300 (Thermo Scientific), coupled online with an Orbitrap Fusion Lumos (Thermo Scientific). Chromatographic separation was performed with a two-column system, consisting of a C-18 trap cartridge (300 µm ID, 5 mm length) and a picofrit analytical column (75 µm ID, 25 cm length) packed in-house with reversed-phase Repro-Sil Pur C18-AQ 3 µm resin. To analyze the proteome, peptides were separated using a 180 min gradient from 4-30% buffer B (buffer A: 0.1% formic acid, buffer B: 80% acetonitrile + 0.1% formic acid) at a flow rate of 300 nl/min. The mass spectrometer was set to acquire spectra in data-dependent acquisition (DDA) mode. The MS scan was set to 300-1200 m/z in the orbitrap with a resolution of 120,000 (at 200 m/z) and an AGC target of 5x10e5. MS/MS was performed in the ion trap using the top speed mode (2 secs), an AGC target of 1x10e4 and an HCD collision energy of 35. To analyze histones, peptides were separated using a 30 min gradient from 1-30% buffer B (buffer A: 0.1% formic acid, buffer B: 80% acetonitrile + 0.1% formic acid) at a flow rate of 300 nl/min. The mass spectrometer was set to acquire spectra in a data-independent acquisition (DIA) mode. The MS scan was set to 300-1100 m/z in the orbitrap with a resolution of 120,000 (at 200 m/z) and an AGC target of 5x10e5. MS/MS was performed in the orbitrap with sequential isolation windows of 50 m/z with an AGC target of 2x10e5 and an HCD collision energy of 30.

Table S1. Gene 0ntologies (GO) identified in the bulk RNA sequencing studies related to viral processes that differentiated HSV-2-infected versus mock-Infected CD4+ T cells.

GO Term	Padj	NES	Size	Name
GO:0019058	0.01456757	-1.6321884	340	viral life cycle
GO:0009615	0.02700039	-1.4266105	340	response to virus
GO:0019080	0.01456757	-2.1579949	195	viral gene expression
GO:1903900	0.01456757	-1.5924113	156	regulation of viral life cycle
GO:0019079	0.01456757	-1.6115278	127	viral genome replication
GO:0048524	0.01456757	-1.6586752	107	positive regulation of viral process
GO:0045069	0.04856415	-1.4683209	100	regulation of viral genome replication
GO:1903902	0.01456757	-1.718878	61	positive regulation of viral life cycle
GO:0019076	0.04051518	-1.6353349	34	viral release from host cell
GO:0050690	0.01456757	-1.9231667	29	regulation of defense response to virus by virus
GO:0046755	0.02436248	-1.7273188	26	viral budding
GO:0039702	0.01480637	-1.7519646	21	viral budding via host ESCRT complex
GO:0019081	0.04759919	-1.6482786	15	viral translation
GO:0030069	0.01560581	-1.8821989	11	viral latency
GO:0075522	0.04465982	-1.6277708	10	IRES-dependent viral translational initiation
GO:0019043	0.01605684	-1.8432589	9	establishment of viral latency

Padj= adjusted p-value comparing GFP+ vs Mock; NES=normalized enrichment score; size= number of transcripts

Table S2: Top 100 genes and their relative contribution to principal component (PC) 1 and PC 2 after performing an analysis of 632 genes in GO:0009615, GO:0019080, and GO:0019058.

PC 1		PC 2	
Gene	Contribution	Gene	Contribution
PCSK5	9.26459536	IFI44L	5.368914383
HSPA1B	1.73059215	IFIT3	4.693099859
HSPA1A	1.46006202	RSAD2	4.517060048
CD74	1.31316537	IFIT1	4.504468024
NUP210	1.2337911	DDX60	3.038212722
FURIN	1.12701999	IFIT2	2.954696673
PPIA	1.07107725	IFI44	2.917818448
GTF2F1	1.06252667	MX1	2.855222521
FBXL2	1.05950698	OAS3	2.583800497
CXADR	0.99650875	OAS1	2.110663427
GRK2	0.92656984	IFIT5	1.719925566
SELPLG	0.86559614	LAMP3	1.490837756
ENO1	0.79920577	MX2	1.462715702
RAB1B	0.79162863	IFI6	1.381562637
UNC13D	0.78059689	TRIM22	1.353476571
IL2RA	0.75819793	PARP10	1.304858458
BANF1	0.72130596	BNIP3	1.139110934
RRP1B	0.7136199	PCSK5	1.100718952
CXCL12	0.70198124	STAT2	1.082835976
CTBP1	0.69712487	BNIP3L	1.007159016
H19	0.69686349	IRF7	0.964603065
STMN1	0.69227627	LGALS9	0.932177717
PHB	0.68403405	DDX58	0.90332716
STING1	0.68093442	ISG20	0.889401553
APOBEC3C	0.66646653	CYP1A1	0.88654098
EPHA2	0.66130569	CCL4	0.851453653
PTPRC	0.65471341	DHX58	0.808275491
ITGB7	0.64687299	HERC5	0.801019769
CFL1	0.63441875	GTF2F1	0.792182168
FADD	0.62722861	SHFL	0.761895213
NDC1	0.62584171	ZBP1	0.718708979
HMGA1	0.61631309	HSPB1	0.716255639
RAN	0.60844204	OAS2	0.712516955
C1QBP	0.60263798	AIM2	0.70954151
PPIB	0.59736833	LGALS1	0.671431504
NMI	0.59419822	OASL	0.658015145
SLC10A1	0.59116349	APOBEC3B	0.64336755
HMGA2	0.58537768	ISG15	0.641545605
SNX3	0.57878812	IFI27	0.637033244
CD28	0.56265531	UBB	0.636197142
BAX	0.55980364	UBC	0.600550319
HSP90AA1	0.55331519	IFNG	0.599661888
CDK1	0.55053431	PARP9	0.592555953
UBC	0.52152225	IRF9	0.511310566
CD4	0.5183775	IKBKE	0.506141672
LGALS1	0.49988468	IFITM1	0.479569443
BATF3	0.4938511	POU2AF1	0.472565458
LY6E	0.48840968	IFIH1	0.470550758
NR5A2	0.48750403	CTSL	0.463671135
ZC3H12A	0.47871598	DTX3L	0.451791927
BCL2L1	0.47368093	ITGB1	0.447315048
BCL2	0.4625831	RTP4	0.44105846
RIOK3	0.45945027	HSPA1A	0.426389951
CCT5	0.45692377	LY6E	0.393396589

EFNB2	0.45544307	PLSCR1	0.383772042
VCP	0.45326924	CD80	0.383152931
NOTCH1	0.44882512	DDX3X	0.3701262
KPNA3	0.44639952	ACTA2	0.351982962
IFITM2	0.44623587	TRIM25	0.348290834
NUP37	0.43594115	EIF2AK2	0.346696898
SMARCB1	0.43066518	TNF	0.343156296
LARP1	0.42529983	STAT1	0.338336794
IFITM1	0.40352407	TRIM56	0.327905431
DPP4	0.40257795	CXADR	0.324197249
PROX1	0.40216639	PSMA2	0.314847892
VAMP8	0.39127688	APOBEC3C	0.309902333
CHMP4B	0.39040608	BSG	0.30819895
MAP3K14	0.38983282	NLRC5	0.303341799
TOP2A	0.38522217	RPS15A	0.302086449
NUP62	0.38009392	CCL5	0.290213881
UBA52	0.37966751	STOM	0.284820123
USP17L2	0.37662308	EEF1G	0.28140068
SLC1A5	0.37638778	TRIM14	0.24667531
TRIM8	0.37268916	CCR5	0.222467598
SPCS1	0.36245356	LAMTOR5	0.218972461
NUCKS1	0.35984206	IL23R	0.211083353
NUP188	0.35965792	GBP1	0.20928792
VPS28	0.35956624	IL21	0.202887021
TNFRSF14	0.3566537	IRF3	0.202073676
LGALS9	0.35554873	UFD1	0.200657387
TRIM14	0.34898783	PML	0.199524885
CAV1	0.34505221	RELA	0.197752109
BST2	0.34279222	RPS27A	0.19426679
HLA-DRB1	0.34214657	HTATSF1	0.193139317
SIVA1	0.33650693	PPIA	0.192204376
CHMP1A	0.32542306	RAN	0.191103571
DDX3X	0.32243912	MOV10	0.190477765
BCL3	0.32033021	CHMP2A	0.188877388
TNFRSF4	0.3199104	CHMP2B	0.183984467
ROCK2	0.31812069	RESF1	0.18334351
SERINC3	0.31661817	IVNS1ABP	0.180206694
PDCD6IP	0.31066215	UNC13D	0.180124511
APOBEC3G	0.30357804	NOTCH1	0.177235546
PRF1	0.30316794	AP1S1	0.169742689
TRIM52	0.30172743	PPIE	0.166995299
PHB2	0.30113912	TBK1	0.166014591
SHFL	0.28955206	CLU	0.160752579
AZU1	0.2881592	TFRC	0.160237583
ADARB1	0.2803953	STMN1	0.159971188
RELA	0.28014466	ZC3HAV1	0.158646571

Table S3: Relative expression of select transcripts associated with HIV reactivation and replication depicted in the volcano plots (Figure 4) comparing HSV-infected (GFP+) versus mock-infected and HSV-exposed but GFP- (bystander cells) versus mock-infected cells.

	GFP+ vs Mock		GFP- vs Mock	
	log2foldchange	padj	log2foldchange	padj
ABCD1	-2.51748	8.65e-015	-0.76948	0.012796
ABI2	0.13931	0.061693	-0.20029	0.010928
ADAM10	-2.55744	1.3e-118	-0.51325	0.00000707
ADAR	-0.93888	7.51e-033	0.107656	0.285397
AGFG1	-0.12786	0.331114	-0.13846	0.377306
AKT1	-2.37376	4.5e-114	-0.48728	0.00000594
APOBEC3F	-0.38792	0.03698	-0.329	0.108752
APOBEC3G	-2.21406	1.01e-098	-0.80106	1.22e-015
APOBEC3H	-1.58811	1.1e-013	-0.67878	0.002031
ATM	-0.27128	0.034957	0.604254	0.00000215
ATR	-0.97522	1.98e-058	-0.11	0.116907
BST2	-2.20707	9.64e-037	-0.50885	0.008463
BTRC	0.102609	0.409283	-0.45545	0.0000872
CCL2	1.921032	0.002171	-1.618	0.052917
CCL4	-2.63655	2.8e-010	-3.2561	1.62e-015
CCL5	-1.39999	2.65e-011	-1.36063	1.91e-010
CCNT1	0.752051	3.81e-026	-0.0949	0.296147
CCR2	-4.54359	2.52e-042	-2.35876	3.59e-017
CCR5	-1.63438	1.61e-021	-1.41667	8.04e-019
CD209	3.472887	1.66e-010	0.948884	0.163704
CD28	-2.73782	6e-227	-1.09538	1.03e-037
CD4	-2.60234	2.8e-203	-0.79151	2.1e-021
CDC25C	-1.72441	8.4e-027	-1.05813	2.92e-012
CLEC4M	5.458343	1.14e-027	0.531438	0.499257
COPB2	-0.03824	0.673376	-0.66946	6.09e-017
CRAT	-3.28633	5.33e-041	-0.90843	1.81e-015
CTDP1	-0.83761	6.41e-013	-0.37384	0.002255
CUL5	0.508694	1.09e-007	-0.15973	0.164405
CX3CR1	-2.08898	0.009048	-1.72695	0.00915
CXCL12	7.372607	6.96e-024	1.322368	0.159504
CXCR4	-0.88338	2.04e-018	-0.60022	7.23e-009
CXCR6	-1.76464	1.68e-043	-0.8392523	8.68e-018
DDX3X	1.699985	5.16e-029	-0.27768	0.136467
DDX5	1.83127667	1.22e-190	0.40149974	9.54e-010
DDX58	0.606028	0.000319	1.211252	4.44e-013
DHX9	-0.53636	6.76e-011	-0.49699	4.33e-009
DMXL1	0.626132	8.54e-014	0.052764	0.651856
DNM2	-1.27274	6.72e-082	-0.26916	0.0000887
DYSF	5.084865	1.23e-007	0.835461	0.55611
EIF2AK2	0.145024	0.489571	0.495938	0.021845
EIF4A2	3.185991	2.5e-300	0.157247	0.059002
ETF1	-1.13	1.05e-030	-0.43468	0.0000326
FBXW11	-0.02456	0.813896	-0.34913	0.000112
FLNB	0.105874	0.242142	-0.90966	1.51e-027
FOS	8.083296	1e-300	1.822632	4.05e-016

GML	4.147984	0.000802	2.346422	0.103655
GOLPH3	-3.28577	1e-213	-0.46844	0.00000375
HGS	-0.92994	2.73e-028	0.065681	0.548413
HTATSF1	-1.17872	1.15e-033	-0.85586	4.88e-018
IDH1	-2.16269	4.29e-091	-1.18479	9.02e-034
IFI35	-1.33218	3.11e-013	0.465036	0.019036
IFI6	-0.45003	0.229407	0.95894	0.01373
IFIH1	0.542244	0.004189	0.742869	0.000152
IFIT1	1.288169	0.00275	2.754199	1.32e-010
IFIT3	2.173164	2.89e-013	3.037048	5.96e-024
IFIT5	0.319352	0.25	1.46724049	4.33e-008
IFITM1	-2.02007	3.77e-040	0.293311	0.104119
IFNAR1	-1.55336	2.1e-136	-0.53047	3.25e-022
IFNAR2	-3.27423	1.6e-112	-0.53284	0.000129
IFNGR1	-0.38674	0.010819	-0.49961	0.001315
IL10	-0.26982	0.40255	-1.30591	0.0000138
IL10RA	-1.5863	1.39e-056	-0.39851	0.0000409
IL12B	1.770856	3.28e-008	0.168626	0.718883
IL18	0.824012	0.018017	0.467734	0.212273
IL1A	-1.55136	0.000213	-2.95295	1.52e-012
IL1B	0.089329	0.859198	-0.52776	0.26732
IL1RN	-1.71302	0.00017	0.572049	0.216054
IL2RA	-3.0488	2.5e-120	-1.00789	7.86e-014
IL32	-4.80367	2.5e-300	-1.46657	5.83e-033
IL4	-0.79013	0.174277	-1.06582	0.046568
IL4R	-2.80879	9.84e-060	0.582143	0.001761
INPP5J	2.035979	0.001187	-0.95039	0.246212
IRF1	-0.20396	0.044189	0.272336	0.010884
IRF7	0.39182	0.117381	1.331416	2.26e-008
IRF9	-0.57271	6.01e-007	0.709116	4.37e-010
ISG15	-0.50558	0.070957	0.751303	0.009833
JAK1	-1.10759	5.45e-049	-0.54622	9.84e-013
JAK2	-0.9448	5.61e-015	-0.10162	0.533444
KHDRBS1	-1.77943	8.59e-094	-0.6938	4.69e-015
KLHDC2	0.328536	0.000316	-0.31083	0.000836
KPNB1	-0.51878	3.92e-014	-0.27932	0.000154
LCK	-3.16269	2.1e-248	-0.46153	0.00000124
LCP2	-2.42301	4.8e-244	0.077778	0.394708
LIG4	0.457999	0.0000111	0.548605	1.48e-007
LPL	2.275088	0.000103	-0.09819	0.925149
LTA	0.113995	0.789296	0.547847	0.231991
LYPD4	3.466887	1.27e-013	0.166903	0.841629
MALAT1	2.65974	4.7e-125	1.103813	1.11e-021
MAP3K5	-0.63742	9.14e-007	-0.73535	1.53e-008
MAP4	-2.34368	2.12e-081	-0.75709	2.42e-009
MED28	-0.73965	2.24e-020	-0.42979	2.29e-007
MED4	-0.84421	1.19e-020	-0.47112	2.88e-007
MED6	-0.68404	3.35e-011	-0.38784	0.000273
MED7	-1.16958	7.27e-051	-0.40182	5.96e-010
MID1IP1	-1.98328	4e-157	-0.51749	3.27e-014
MX1	0.160337	0.72051	1.326196	0.002037

MX2	-0.82938	0.00026	1.092461	0.00000255
NEDD4L	1.667592	8.61e-014	0.107147	0.756847
NMT1	-0.52794	0.00000551	-0.62507	1.14e-007
NUP153	-1.29721	4.53e-081	0.029144	0.781932
NUP85	-0.49364	7.22e-012	-0.25174	0.000688
OAS1	-0.77161	0.03927	1.123705	0.004274
OAS2	-1.54581	4.48e-012	0.360195	0.191166
OAS3	0.131296	0.649849	1.626718	6.76e-010
OASL	-0.02956	0.904841	0.885854	0.0000464
PAK2	-2.49586	6.7e-146	-0.42887	0.0000295
PCSK5	11.42827	2.5e-300	4.576987	9.9e-156
PDCD6IP	-1.90979	1.45e-082	-0.2877	0.008714
PDIA6	-2.59366	1.4e-126	-1.02838	7.19e-021
PIAS4	-2.55187	3.03e-040	-0.16914	0.486272
PML	-1.02673	2.59e-021	0.270452	0.026096
PPIA	-3.66194	1e-300	-1.35107	1.81e-042
PRF1	-4.04329	1.59e-041	-1.23686	3.77e-007
PSIP1	-1.23801	2.11e-093	-0.56681	9.73e-021
PSMB8	-3.27565	5.4e-171	-0.72067	1.53e-009
PTPN2	-1.15415	1.94e-049	-0.49951	1.57e-010
PTPRC	-2.72789	4.5e-273	-0.58022	2.94e-013
RANBP1	-2.26947	2.4e-122	-0.86022	1.38e-018
RANBP2	-1.31679	8.19e-090	-0.34805	3.91e-007
RELA	-1.85355	5.6e-116	0.24566	0.001226
RGP1	-1.01327	1.21e-054	-0.02227	0.803261
RNASEL	-0.47685	0.000679	0.356824	0.009781
RSAD2	0.068	0.82	2.697459	6.41e-025
SERINC3	-2.1210324	8.47e-165	-0.76489	5.69e-025
SLC2A1	-2.28761	3.3e-079	-0.43159	0.000153
SMARCB1	-2.51702	5.09e-074	-0.65855	0.00000354
SOCS1	-1.56879	1.29e-016	1.001707	2.99e-008
SPTAN1	0.561189	0.000184	-0.27951	0.110089
STAT1	-0.91712	3.49e-016	0.399739	0.001198
STAT2	0.142181	0.381642	1.185135	3.21e-015
STAU1	-1.27517	1.62e-051	-0.41779	0.00000216
STAU2	-1.65918	2.39e-043	-0.49595	0.000059
TAP1	-2.92354	4.3e-150	-0.09015	0.561173
THOC2	-0.84097	5.57e-036	-0.42566	5.7e-010
TLR7	1.402633	0.002685	1.564884	0.000254
TLR8	1.892337	0.018892	0.415941	0.710968
TLR9	2.14187	2.35e-007	0.788743	0.103304
TNF	1.94	7.93e-019	1.39	9.24e-010
TNFRSF1A	-1.89304	2.31e-054	-0.18351	0.124008
TNPO3	-0.31352	1.5e-007	-0.51432	2.82e-018
TRAPPC1	-3.41936	3.3e-199	-1.19734	6.28e-039
TRIM22	-0.0072	0.97793	1.152389	1.08e-007
TRIM32	-0.85843	1.1e-016	0.061953	0.640087
TRIM5	-0.03208	0.865455	0.222104	0.268954
TRIM55	4.56458	2e-008	1.047635	0.340324
TSG101	-1.26817	9.06e-045	-0.44461	1.1e-007
TYK2	-1.14689	1.47e-039	0.08538	0.441188

UBE2I	-2.04236	4.7e-151	-0.6484	3.09e-017
UNG	-3.60575	6.3e-266	-0.89772	3.26e-023
VDR	-2.51791	7.05e-095	-1.69579	3.2e-045
VPS4A	-1.73382	5.67e-082	-0.12788	0.231596
XPO1	0.144579	0.014724	-0.24713	0.000041
XRCC5	-1.81416	5.86e-094	-0.83396	3.3e-020
ZBP1	0.613243	0.004619	1.847528	8.14e-022
ZNF436	-0.23832	0.021078	0.385811	0.0000446
ZNF536	6.907543	3.27e-017	3.731298	0.0000302

Table S5. Histone peptide analysis of Empty mCherry vector (control) and VP16 transfected 2D10 cells. Values are relative abundances of the peptides normalized by the same peptide sequence in all its modified states.

Peptide	Empty vector	VP16	log2 fold change	Neg log2 p-value
TKQTAR(H3_3_8)				
H3_3_8 unmod	88.24%	70.05%	-0.18	3.44
H3_3_8 K4me1	11.76%	29.95%	0.18	3.44
H3_3_8 K4me2	0.00%	0.00%	0.00	
H3_3_8 K4me3	0.00%	0.00%	0.00	
H3_3_8 K4ac	0.00%	0.00%	0.00	
KSTGGKAPR(H3_9_17)				
H3_9_17 unmod	24.55%	17.14%	-0.07	6.34
H3_9_17 K9me1	8.37%	8.77%	0.00	0.10
H3_9_17 K9me2	6.97%	13.06%	0.06	3.21
H3_9_17 K9me3	5.66%	7.04%	0.01	4.66
H3_9_17 K9ac	2.20%	0.50%	-0.02	3.82
H3_9_17 K14ac	28.74%	16.26%	-0.12	5.49
H3_9_17 K9me1K14ac	11.59%	6.18%	-0.05	5.48
H3_9_17 K9me2K14ac	4.61%	9.00%	0.04	6.04
H3_9_17 K9me3K14ac	2.26%	3.52%	0.01	3.40
H3_9_17 K9acK14ac	5.05%	18.52%	0.13	3.52
KQLATKAAR(H3_18_26)				
H3_18_26 unmod	55.97%	54.47%	-0.01	0.58
H3_18_26 K23me1	0.00%	0.03%	0.00	1.22
H3_18_26 K18me1	0.01%	0.02%	0.00	0.83
H3_18_26 K18me1K23me1	0.00%	0.00%	0.00	
H3_18_26 K18ac	2.96%	1.72%	-0.01	1.62
H3_18_26 K23ac	39.99%	42.85%	0.03	1.10
H3_18_26 K18acK23ac	1.07%	0.92%	0.00	0.73
KSAPATGGVKKPHR(H3_27_40)				
H3_27_40 unmod	2.48%	1.99%	0.00	0.37
H3_27_40 K36me1	0.37%	0.14%	0.00	3.68
H3_27_40 K27me1	5.46%	2.32%	-0.03	4.49
H3_27_40 K27me2	34.68%	32.22%	-0.02	3.03
H3_27_40 K36me2	2.76%	3.76%	0.01	5.42
H3_27_40 K27me3	15.73%	10.76%	-0.05	4.90
H3_27_40 K36me3	1.05%	0.38%	-0.01	2.16
H3_27_40 K27me2K36me1	13.75%	16.75%	0.03	3.68
H3_27_40 K27me1K36me2	6.44%	9.22%	0.03	5.90
H3_27_40 K27me1K36me1	0.54%	0.17%	0.00	1.68
H3_27_40 K27me3K36me1	3.51%	3.57%	0.00	0.17
H3_27_40 K27me1K36me3	0.28%	0.00%	0.00	1.57
H3_27_40 K27me2K36me2	10.88%	16.38%	0.06	3.61
H3_27_40 K27me3K36me2	2.06%	2.32%	0.00	0.24
H3_27_40 K27ac	0.00%	0.00%	0.00	
KSAPSTGGVKKPHR(H33_27_40)				
H33_27_40 unmod	0.00%	0.00%	0.00	
H33_27_40 K36me1	0.00%	0.00%	0.00	
H33_27_40 K27me1	0.00%	0.00%	0.00	
H33_27_40 K27me2	27.24%	25.21%	-0.02	0.11
H33_27_40 K36me2	34.64%	0.00%	-0.35	2.16
H33_27_40 K27me3	5.59%	0.00%	-0.06	1.63
H33_27_40 K36me3	1.68%	28.28%	0.27	2.09
H33_27_40 K27me2K36me1	22.09%	46.50%	0.24	1.78
H33_27_40 K27me1K36me2	0.00%	0.00%	0.00	
H33_27_40 K27me1K36me1	0.00%	0.00%	0.00	
H33_27_40 K27me3K36me1	0.00%	0.00%	0.00	
H33_27_40 K27me1K36me3	0.00%	0.00%	0.00	
H33_27_40 K27me2K36me2	8.76%	0.00%	-0.09	2.21
H33_27_40 K27me3K36me2	0.00%	0.00%	0.00	
H33_27_40 K27ac	0.00%	0.00%	0.00	

YQKSTELLIR(H3_54_63)				
H3_54_63 unmod	62.37%	66.31%	0.04	0.34
H3_54_63 K56me1	6.37%	12.14%	0.06	0.99
H3_54_63 K56me2	0.00%	0.00%	0.00	
H3_54_63 K56me3	0.00%	0.33%	0.00	1.24
H3_54_63 K56ac	31.27%	21.21%	-0.10	1.17
EIAQDFKTDLR(H3_73_83)				
H3_73_83 unmod	26.16%	28.91%	0.03	0.79
H3_73_83 K79me1	0.12%	0.15%	0.00	0.19
H3_73_83 K79me2	71.60%	70.30%	-0.01	0.47
H3_73_83 K79me3	2.12%	0.64%	-0.01	1.57
H3_73_83 K79ac	0.00%	0.00%	0.00	
VTIMPKDIQLAR(H3_117_128)				
H3_117_128 unmod	100.00%	100.00%	0.00	
H3_117_128 K122ac	0.00%	0.00%	0.00	
GKGGKGLGKGGAKR(H4_4_17)				
H4_4_17 unmod	69.08%	67.86%	-0.01	0.36
H4_4_17 K5ac	5.53%	2.19%	-0.03	2.14
H4_4_17 K8ac	5.53%	2.04%	-0.03	2.16
H4_4_17 K12ac	5.53%	6.15%	0.01	1.23
H4_4_17 K16ac	5.53%	14.49%	0.09	2.59
H4_4_17 K5acK8ac	1.27%	0.49%	-0.01	2.91
H4_4_17 K5acK12ac	1.27%	0.38%	-0.01	2.97
H4_4_17 K5acK16ac	1.27%	1.79%	0.01	1.12
H4_4_17 K8acK12ac	1.27%	0.64%	-0.01	2.66
H4_4_17 K8acK16ac	1.27%	0.41%	-0.01	2.98
H4_4_17 K12acK16ac	1.27%	3.06%	0.02	1.81
H4_4_17 K5acK8acK12ac	0.27%	0.21%	0.00	0.96
H4_4_17 K5acK8acK16ac	0.27%	0.10%	0.00	3.86
H4_4_17 K5acK12acK16ac	0.27%	0.10%	0.00	3.83
H4_4_17 K8acK12acK16ac	0.27%	0.10%	0.00	3.83
H4_4_17 K5acK8acK12acK16ac	0.10%	0.00%	0.00	2.24
KVLR(H4_20_23)				
H4_20_23 unmod	23.14%	8.95%	-0.14	4.53
H4_20_23 K20me1	51.17%	35.48%	-0.16	10.36
H4_20_23 K20me2	0.00%	0.00%	0.00	
H4_20_23 K20me3	25.69%	55.57%	0.30	7.50
H4_20_23 K20ac	0.00%	0.00%	0.00	
RGGVKR(H4_40_45)				
H4_40_45 unmod	100.00%	99.87%	0.00	1.97
H4_40_45 K44ac	0.00%	0.13%	0.00	1.97
KSAGAAKR(H14_25_32)				
H14_25_32 unmod	7.64%	8.07%	0.00	1.89
H14_25_32 K25me1	0.98%	0.24%	-0.01	1.54
H14_25_32 K25me2	0.00%	0.00%	0.00	
H14_25_32 K25me3	0.00%	0.00%	0.00	
H14_25_32 K25ac	0.00%	0.00%	0.00	
H14_25_32 K31ac	0.00%	0.00%	0.00	
H14_25_32 S26ac	3.15%	3.08%	0.00	0.03
H14_25_32 S26ph	88.23%	88.60%	0.00	0.17
KASGPPVSELITKAVAASKER(H12_33_53)				
H12_33_53 unmod	68.03%	83.78%	0.16	1.33
H12_33_53 K33me1	21.17%	10.90%	-0.10	0.76
H12_33_53 K33me2	0.32%	0.00%	0.00	1.24
H12_33_53 K33me3	0.00%	0.00%	0.00	
H12_33_53 K33ac	10.47%	0.00%	-0.10	1.24
H12_33_53 S40ac	0.00%	5.32%	0.05	1.24
KATGPPVSELITKAVAASKER(H15_36_56)				
H15_36_56 unmod	24.92%	10.99%	-0.14	1.79
H15_36_56 K36me1	0.88%	1.12%	0.00	0.19
H15_36_56 K36me2	0.00%	0.00%	0.00	
H15_36_56 K36me3	15.40%	5.68%	-0.10	0.75
H15_36_56 K36ac	51.51%	80.25%	0.29	1.32
H15_36_56 S43ac	7.29%	1.95%	-0.05	1.04
unmod(H1_1_35)				

H1_1_35 H12.SETAPAAPAAAPPAEKAPVKKKAAKKAGGTPR	15.81%	12.84%	-0.03	0.36
H1_1_35 H13.SETAPLAPTIPAPAEKTPVKKKAKKAGATAGKR	42.07%	55.16%	0.13	0.48
H1_1_35 H14.SETAPAAPAAPAPAEKTPVKKKAR	11.37%	3.10%	-0.08	1.63
H1_1_35 H15.SETAPAETATPAPVEKSPAKKKATKKAAGAGAAKR	30.74%	28.90%	-0.02	0.09
unmod(H1_54_81)				
H1_54_81 H11.GGVSLAALKKALAAAGYDVEKNNSR	0.00%	0.00%	0.00	
H1_54_81 H1v234.SGVSLAALKKALAAAGYDVEKNNSR	6.32%	59.62%	0.53	2.19
H1_54_81 H15.NGLSLAALKKALAAGGYDVEKNNSR	0.00%	0.00%	0.00	
H1_54_81 H1T.VGMSLVALKKALAAAGYDVEKNNSR	60.35%	7.05%	-0.53	2.18
KGNYAER(H2A1_36_42)				
H2A1_36_42 unmod	76.81%	51.94%	-0.25	10.23
H2A1_36_42 K36ac	22.47%	48.01%	0.26	11.04
H2A1_36_42 Y39ac	0.72%	0.05%	-0.01	2.44
KGNYSER(H2A3_36_42)				
H2A3_36_42 unmod	93.10%	92.96%	0.00	0.22
H2A3_36_42 K36ac	6.19%	5.71%	0.00	3.08
H2A3_36_42 Y39ac	0.71%	1.34%	0.01	1.21
KGHYAER(H2AX_36_42)				
H2AX_36_42 unmod	33.94%	56.81%	0.23	3.70
H2AX_36_42 K36ac	66.06%	14.11%	-0.52	4.43
H2AX_36_42 Y39ac	0.00%	29.08%	0.29	2.39
GKQGGKAR(H2A1_4_11)				
H2A1_4_11 unmod	97.50%	94.06%	-0.03	3.77
H2A1_4_11 K5ac	0.15%	0.68%	0.01	2.53
H2A1_4_11 K9ac	2.01%	3.14%	0.01	2.16
H2A1_4_11 K5acK9ac	0.07%	0.24%	0.00	1.19
H2A1_4_11 K9me1	0.02%	0.02%	0.00	0.12
H2A1_4_11 K5me1	0.25%	1.87%	0.02	1.53
GKQGGKVR(H2AJ_4_11)				
H2AJ_4_11 unmod	4.68%	0.43%	-0.04	1.55
H2AJ_4_11 K5ac	0.04%	0.35%	0.00	1.09
H2AJ_4_11 K9ac	0.57%	6.19%	0.06	1.87
H2AJ_4_11 K5acK9ac	62.02%	57.42%	-0.05	0.12
H2AJ_4_11 K9me1	2.35%	1.89%	0.00	0.19
H2AJ_4_11 K5me1	30.35%	33.73%	0.03	0.11
GKTGGKAR(H2AX_4_11)				
H2AX_4_11 unmod	86.22%	86.05%	0.00	0.10
H2AX_4_11 K5ac	0.84%	1.01%	0.00	0.75
H2AX_4_11 K9ac	11.36%	11.12%	0.00	0.43
H2AX_4_11 K5acK9ac	1.27%	1.26%	0.00	0.02
H2AX_4_11 K9me1	0.02%	0.00%	0.00	1.24
H2AX_4_11 K5me1	0.29%	0.56%	0.00	0.93
SGRGKQGGKAR(H2A1_1_11)				
H2A1_1_11 unmod	0.85%	1.64%	0.01	1.20
H2A1_1_11 S1ac	52.73%	35.49%	-0.17	1.20
H2A1_1_11 K5ac	46.42%	62.87%	0.16	1.20
AGGKAGKDSGKAKAKAVSR(H2AV_1_19)				
H2AV_1_19 unmod	94.91%	79.83%	-0.15	0.91
H2AV_1_19 K4ac	0.00%	0.00%	0.00	
H2AV_1_19 K7ac	0.00%	0.00%	0.00	
H2AV_1_19 K11ac	0.00%	0.00%	0.00	
H2AV_1_19 K15ac	0.00%	0.00%	0.00	
H2AV_1_19 K4acK7ac	0.57%	0.00%	-0.01	1.24
H2AV_1_19 K4acK11ac	0.57%	0.00%	-0.01	1.24
H2AV_1_19 K4acK15ac	0.57%	0.00%	-0.01	1.24
H2AV_1_19 K7acK11ac	0.57%	0.00%	-0.01	1.24
H2AV_1_19 K7acK15ac	0.57%	0.00%	-0.01	1.24
H2AV_1_19 K11acK15ac	0.57%	0.00%	-0.01	1.24
H2AV_1_19 K7acK11acK15ac	0.42%	0.00%	0.00	1.24
H2AV_1_19 K4acK11acK15ac	0.42%	0.00%	0.00	1.24
H2AV_1_19 K4acK7acK15ac	0.42%	0.00%	0.00	1.24
H2AV_1_19 K4acK7acK11ac	0.42%	0.00%	0.00	1.24
H2AV_1_19 K4acK7acK11acK15ac	0.00%	20.17%	0.20	1.24
AGGKAGKDSGKAKTKAVSR(H2AZ_1_19)				

H2AZ_1_19 unmod	63.77%	35.54%	-0.28	1.93
H2AZ_1_19 K4ac	5.50%	9.09%	0.04	0.86
H2AZ_1_19 K7ac	5.50%	9.09%	0.04	0.86
H2AZ_1_19 K11ac	5.50%	9.19%	0.04	0.90
H2AZ_1_19 K15ac	5.50%	18.48%	0.13	3.02
H2AZ_1_19 K4acK7ac	0.01%	0.08%	0.00	1.03
H2AZ_1_19 K4acK11ac	0.01%	0.08%	0.00	1.03
H2AZ_1_19 K4acK15ac	0.01%	0.08%	0.00	1.03
H2AZ_1_19 K7acK11ac	0.01%	0.08%	0.00	1.03
H2AZ_1_19 K7acK15ac	0.01%	0.08%	0.00	1.03
H2AZ_1_19 K11acK15ac	0.01%	0.08%	0.00	1.03
H2AZ_1_19 K7acK11acK15ac	3.54%	0.59%	-0.03	3.37
H2AZ_1_19 K4acK11acK15ac	3.54%	0.59%	-0.03	3.37
H2AZ_1_19 K4acK7acK15ac	3.54%	0.75%	-0.03	3.19
H2AZ_1_19 K4acK7acK11ac	3.54%	16.20%	0.13	1.01
H2AZ_1_19 K4acK7acK11acK15ac	0.00%	0.00%	0.00	
AKAKTR(H2A1_12_17)				
H2A1_12_17 unmod	31.15%	36.43%	0.05	1.56
H2A1_12_17 K13ac	0.09%	0.01%	0.00	7.77
H2A1_12_17 K15ac	1.20%	0.53%	-0.01	3.94
H2A1_12_17 K15me1	4.74%	42.48%	0.38	2.44
H2A1_12_17 K13me1	62.40%	20.49%	-0.42	2.59
H2A1_12_17 T16ac	0.42%	0.06%	0.00	8.70
AKAKSR(H2A3_12_17)				
H2A3_12_17 unmod	3.82%	1.25%	-0.03	5.81
H2A3_12_17 K13ac	0.01%	0.00%	0.00	1.63
H2A3_12_17 K15ac	0.18%	0.08%	0.00	0.78
H2A3_12_17 K15me1	6.84%	25.19%	0.18	1.05
H2A3_12_17 K13me1	89.15%	73.48%	-0.16	0.86
H2A3_12_17 S16ac	0.00%	0.00%	0.00	
DNKKTR(H2A1_72_77)				
H2A1_72_77 unmod	91.92%	80.85%	-0.11	6.09
H2A1_72_77 K74ac	8.08%	19.15%	0.11	6.09
unmod(H2A_1_88)				
H2A_1_88 H2A14s.HLQLAIR	74.88%	79.11%	0.04	0.55
H2A_1_88 H2AZ.AGGKAGKDSGKAKTKAVSR	18.27%	12.09%	-0.06	0.74
H2A_1_88 H2AY.SAKAGVIFPVGR	0.09%	0.24%	0.00	3.33
H2A_1_88 H2AX.GKTGGKAR	6.76%	8.55%	0.02	1.64
LAHYNKR(H2B1B_80_86)				
H2B1B_80_86 unmod	91.06%	93.19%	0.02	0.90
H2B1B_80_86 Y83ac	8.94%	6.81%	-0.02	0.90
LAHYSKR(H2B1A_81_87)				
H2B1A_81_87 unmod	82.98%	81.39%	-0.02	2.81
H2B1A_81_87 K86ac	17.02%	18.61%	0.02	2.81
unmod(H2B_1_29)				
H2B_1_29 1C.PEPAKSAPAPKKGSKKAVTKAQKKDGKKR	31.38%	15.34%	-0.16	3.64
H2B_1_29 1H.PDPAKSAPAPKKGSKKAVTKAQKKDGKKR	20.65%	44.55%	0.24	1.11
H2B_1_29 2F.PDPAKSAPAPKKGSKKAVTKVQKKDGKKR	3.48%	4.99%	0.02	0.29
H2B_1_29 1B.PEPSKSAPAPKKGSKKAITKAQKKDGKKR	14.83%	32.83%	0.18	1.26
H2B_1_29 1N.PEPSKSAPAPKKGSKKAVTKAQKKDGKKR	0.00%	0.00%	0.00	
H2B_1_29 1D.PEPTKSAPAPKKGSKKAVTKAQKKDGKKR	0.00%	0.00%	0.00	
H2B_1_29 1M.PEPVKSAPVPKKGSKKAINKAQKKDGKKR	6.63%	2.29%	-0.04	0.76
H2B_1_29 1L.PELAKSAPAPKKGSKKAVTKAQKKDGKKR	23.03%	0.00%	-0.23	2.41

Table S6. Histone peptide analysis of control vs HSV-2 infected cells at 24h. Values are relative abundances of the peptides normalized by the same peptide sequence in all its modified states.

Peptide	NT	HSV	log2 fold change	Neg log2 p-value
TKQTAR(H3_3_8)				
H3_3_8 unmod	85.84%	83.04%	-0.03	5.00
H3_3_8 K4me1	14.04%	16.81%	0.03	4.76
H3_3_8 K4me2	0.08%	0.01%	0.00	3.20
H3_3_8 K4me3	0.02%	0.01%	0.00	0.87
H3_3_8 K4ac	0.02%	0.12%	0.00	3.41
KSTGGKAPR(H3_9_17)				
H3_9_17 unmod	35.30%	20.53%	-0.15	4.01
H3_9_17 K9me1	9.11%	3.89%	-0.05	6.13
H3_9_17 K9me2	6.82%	6.96%	0.00	0.47
H3_9_17 K9me3	6.06%	5.98%	0.00	0.21
H3_9_17 K9ac	0.09%	0.11%	0.00	0.80
H3_9_17 K14ac	29.86%	39.21%	0.09	5.89
H3_9_17 K9me1K14ac	7.66%	13.65%	0.06	5.91
H3_9_17 K9me2K14ac	3.45%	6.14%	0.03	4.24
H3_9_17 K9me3K14ac	1.37%	2.90%	0.02	6.75
H3_9_17 K9acK14ac	0.24%	0.44%	0.00	3.21
KSTGGKAPR(H3_9_17)				
H3_9_17 S10ph	0.01%	0.04%	0.00	1.00
H3_9_17 K9me1S10ph	0.00%	0.01%	0.00	0.79
H3_9_17 K9me2S10ph	0.00%	0.04%	0.00	1.00
H3_9_17 K9me3S10ph	0.01%	0.05%	0.00	1.12
H3_9_17 K9acS10ph	0.00%	0.00%	0.00	1.00
H3_9_17 S10phK14ac	0.00%	0.00%	0.00	6.67
H3_9_17 K9me1S10phK14ac	0.02%	0.01%	0.00	0.94
H3_9_17 K9me2S10phK14ac	0.00%	0.03%	0.00	1.27
H3_9_17 K9me3S10phK14ac	0.00%	0.02%	0.00	1.00
H3_9_17 K9acS10phK14ac	0.00%	0.00%	0.00	1.00
KQLATKAAR(H3_18_26)				
H3_18_26 unmod	71.78%	63.67%	-0.08	5.78
H3_18_26 K23me1	0.04%	0.00%	0.00	1.42
H3_18_26 K18me1	0.03%	0.00%	0.00	1.04
H3_18_26 K18me1K23me1	0.17%	0.01%	0.00	0.98
H3_18_26 K18ac	1.11%	0.95%	0.00	3.06
H3_18_26 K23ac	25.92%	34.16%	0.08	4.99
H3_18_26 K18acK23ac	0.95%	1.21%	0.00	3.57
KSAPATGGVKKPHR(H3_27_40)				
H3_27_40 unmod	5.82%	2.74%	-0.03	6.41
H3_27_40 K36me1	1.99%	1.27%	-0.01	1.40
H3_27_40 K27me1	6.26%	5.31%	-0.01	3.99
H3_27_40 K27me2	35.40%	33.18%	-0.02	5.05
H3_27_40 K36me2	2.06%	2.29%	0.00	1.53
H3_27_40 K27me3	17.58%	18.93%	0.01	3.14
H3_27_40 K36me3	4.46%	6.86%	0.02	4.89
H3_27_40 K27me2K36me1	5.28%	3.87%	-0.01	1.84
H3_27_40 K27me1K36me2	4.29%	5.48%	0.01	3.05
H3_27_40 K27me1K36me1	1.96%	2.41%	0.00	2.31
H3_27_40 K27me3K36me1	2.82%	3.56%	0.01	3.20
H3_27_40 K27me1K36me3	1.90%	1.48%	0.00	2.31
H3_27_40 K27me2K36me2	7.99%	10.16%	0.02	1.77
H3_27_40 K27me3K36me2	2.03%	2.37%	0.00	1.50
H3_27_40 K27ac	0.07%	0.00%	0.00	7.02
KSAPATGGVKKPHR(H3_27_40)				
H3_27_40 S28ph	0.06%	0.02%	0.00	0.72
H3_27_40 K27me1S28ph	0.01%	0.04%	0.00	1.69
H3_27_40 K27me2S28ph	0.00%	0.03%	0.00	1.78

H3_27_40 K27me3S28ph	0.00%	0.02%	0.00	1.36
KSAPSTGGVKKPHR(H33_27_40)				
H33_27_40 unmod	11.28%	0.35%	-0.11	1.93
H33_27_40 K36me1	3.41%	0.00%	-0.03	2.79
H33_27_40 K27me1	3.05%	0.00%	-0.03	1.27
H33_27_40 K27me2	22.77%	0.03%	-0.23	3.93
H33_27_40 K36me2	6.10%	3.14%	-0.03	0.97
H33_27_40 K27me3	9.31%	0.00%	-0.09	6.24
H33_27_40 K36me3	5.37%	0.00%	-0.05	2.57
H33_27_40 K27me2K36me1	9.99%	0.00%	-0.10	4.78
H33_27_40 K27me1K36me2	6.97%	34.15%	0.27	0.81
H33_27_40 K27me1K36me1	2.08%	0.00%	-0.02	2.68
H33_27_40 K27me3K36me1	2.45%	0.00%	-0.02	3.75
H33_27_40 K27me1K36me3	3.32%	6.61%	0.03	0.50
H33_27_40 K27me2K36me2	11.78%	0.00%	-0.12	4.55
H33_27_40 K27me3K36me2	1.13%	0.00%	-0.01	7.67
H33_27_40 K27ac	0.01%	0.00%	0.00	1.00
KSAPSTGGVKKPHR(H33_27_40)				
H33_27_40 S28ph	0.93%	5.49%	0.05	0.84
H33_27_40 K27me1S28ph	0.02%	50.07%	0.50	1.00
H33_27_40 K27me2S28ph	0.02%	0.05%	0.00	0.63
H33_27_40 K27me3S28ph	0.01%	0.10%	0.00	0.88
YRPGTVALR(H3_41_49)				
H3_41_49 unmod	99.96%	99.99%	0.00	2.35
H3_41_49 Y41ph	0.04%	0.01%	0.00	2.35
YQKSTELLIR(H3_54_63)				
H3_54_63 unmod	94.02%	99.57%	0.06	1.43
H3_54_63 K56me1	0.23%	0.04%	0.00	0.81
H3_54_63 K56me2	4.00%	0.21%	-0.04	1.91
H3_54_63 K56me3	0.00%	0.00%	0.00	
H3_54_63 K56ac	1.76%	0.17%	-0.02	0.92
EIAQDFKTDLR(H3_73_83)				
H3_73_83 unmod	86.06%	90.03%	0.04	1.06
H3_73_83 K79me1	6.74%	4.48%	-0.02	1.08
H3_73_83 K79me2	7.04%	5.35%	-0.02	1.10
H3_73_83 K79me3	0.11%	0.07%	0.00	1.09
H3_73_83 K79ac	0.05%	0.07%	0.00	1.66
VTIMPKDIQLAR(H3_117_128)				
H3_117_128 unmod	84.52%	88.16%	0.04	0.55
H3_117_128 K122ac	15.48%	11.84%	-0.04	0.55
GKGGKGLGKGGAKR(H4_4_17)				
H4_4_17 unmod	62.24%	52.27%	-0.10	7.27
H4_4_17 K5ac	1.13%	1.37%	0.00	1.61
H4_4_17 K8ac	1.98%	2.70%	0.01	3.33
H4_4_17 K12ac	3.02%	6.37%	0.03	8.61
H4_4_17 K16ac	23.62%	23.81%	0.00	0.48
H4_4_17 K5acK8ac	0.20%	0.37%	0.00	3.32
H4_4_17 K5acK12ac	0.50%	0.54%	0.00	1.18
H4_4_17 K5acK16ac	0.48%	0.55%	0.00	1.01
H4_4_17 K8acK12ac	0.45%	1.28%	0.01	2.45
H4_4_17 K8acK16ac	1.47%	2.35%	0.01	3.84
H4_4_17 K12acK16ac	3.31%	5.12%	0.02	2.71
H4_4_17 K5acK8acK12ac	0.19%	0.61%	0.00	1.12
H4_4_17 K5acK8acK16ac	0.26%	0.38%	0.00	1.51
H4_4_17 K5acK12acK16ac	0.13%	0.67%	0.01	1.27
H4_4_17 K8acK12acK16ac	0.76%	1.19%	0.00	2.30
H4_4_17 K5acK8acK12acK16ac	0.24%	0.42%	0.00	2.54
KVLR(H4_20_23)				
H4_20_23 unmod	46.36%	62.58%	0.16	3.21
H4_20_23 K20me1	46.07%	29.31%	-0.17	3.46
H4_20_23 K20me2	5.70%	5.23%	0.00	1.10
H4_20_23 K20me3	1.32%	1.96%	0.01	2.87
H4_20_23 K20ac	0.54%	0.93%	0.00	0.49
DNIQGITKPAIR(H4_24_35)				
H4_24_35 unmod	100.00%	100.00%	0.00	

RGGVKR(H4_40_45)				
H4_40_45 unmod	99.90%	99.98%	0.00	1.68
H4_40_45 K44ac	0.10%	0.02%	0.00	1.68
KTVTAMDVVYALKR(H4_79_92)				
H4_79_92 unmod	100.00%	100.00%	0.00	
unmod(H1_1_35)				
H1_1_35 H12.SETAPAAPAAAPPAEKAPVKKKAAKKAGGTPR	2.63%	3.07%	0.00	1.09
H1_1_35 H13.SETAPLAPTIPAPAEKTPVKKKAKKAGATAGKR	3.23%	8.53%	0.05	1.48
H1_1_35 H14.SETAPAAPAAPAPAEKTPVKKKAR	80.46%	80.68%	0.00	0.02
H1_1_35 H15.SETAPAETATPAPVEKSPAKKKATKKAAGAGAAKR	13.68%	7.72%	-0.06	0.48
unmod(H1_54_81)				
H1_54_81 H11.GGVSLAALKKALAAAGYDVEKNNSR	41.82%	43.06%	0.01	0.08
H1_54_81 H1v234.SGVSLAALKKALAAAGYDVEKNNSR	39.61%	43.88%	0.04	0.31
H1_54_81 H15.NGLSLAALKKALAAGGYDVEKNNSR	18.50%	12.90%	-0.06	1.50
H1_54_81 H1T.VGMSLVALKKALAAAGYDVEKNNSR	0.07%	0.16%	0.00	2.09
KGNYAER(H2A1_36_42)				
H2A1_36_42 unmod	99.99%	99.97%	0.00	0.95
H2A1_36_42 K36ac	0.01%	0.03%	0.00	0.95
KGNYSER(H2A3_36_42)				
H2A3_36_42 unmod	99.45%	99.34%	0.00	0.30
H2A3_36_42 K36ac	0.55%	0.66%	0.00	0.30
KGHYAER(H2AX_36_42)				
H2AX_36_42 unmod	98.77%	99.43%	0.01	0.89
H2AX_36_42 K36ac	1.23%	0.57%	-0.01	0.89
GKQGGKAR(H2A1_4_11)				
H2A1_4_11 unmod	96.62%	97.47%	0.01	1.34
H2A1_4_11 K5ac	2.53%	1.23%	-0.01	8.15
H2A1_4_11 K9ac	0.53%	0.48%	0.00	2.30
H2A1_4_11 K5acK9ac	0.04%	0.11%	0.00	5.20
H2A1_4_11 K9me1	0.16%	0.66%	0.01	0.95
H2A1_4_11 K5me1	0.12%	0.05%	0.00	0.79
GKQGGKVR(H2AJ_4_11)				
H2AJ_4_11 unmod	1.17%	2.25%	0.01	0.98
H2AJ_4_11 K5ac	0.08%	0.27%	0.00	0.89
H2AJ_4_11 K9ac	0.18%	0.35%	0.00	0.62
H2AJ_4_11 K5acK9ac	95.61%	93.50%	-0.02	0.70
H2AJ_4_11 K9me1	0.43%	0.95%	0.01	1.16
H2AJ_4_11 K5me1	2.54%	2.69%	0.00	0.13
GKTGGKAR(H2AX_4_11)				
H2AX_4_11 unmod	9.03%	7.29%	-0.02	0.34
H2AX_4_11 K5ac	0.73%	1.56%	0.01	3.11
H2AX_4_11 K9ac	0.54%	1.45%	0.01	2.27
H2AX_4_11 K5acK9ac	0.02%	0.01%	0.00	0.64
H2AX_4_11 K9me1	66.72%	66.17%	-0.01	0.12
H2AX_4_11 K5me1	22.96%	23.53%	0.01	0.64
SGRGKQGGKAR(H2A1_1_11)				
H2A1_1_11 unmod	79.69%	2.46%	-0.77	3.78
H2A1_1_11 S1ac	12.09%	96.00%	0.84	3.53
H2A1_1_11 K5ac	8.22%	1.54%	-0.07	2.33
AGGKAGKDSGKAKAKAVSR(H2AV_1_19)				
H2AV_1_19 unmod	88.75%	90.99%	0.02	1.19
H2AV_1_19 K4ac	1.77%	1.90%	0.00	0.10
H2AV_1_19 K7ac	1.33%	0.91%	0.00	0.47
H2AV_1_19 K11ac	2.06%	0.14%	-0.02	2.24
H2AV_1_19 K15ac	1.98%	0.95%	-0.01	1.10
H2AV_1_19 K4acK7ac	0.13%	0.03%	0.00	0.79
H2AV_1_19 K4acK11ac	0.11%	0.06%	0.00	0.48
H2AV_1_19 K4acK15ac	0.00%	0.00%	0.00	
H2AV_1_19 K7acK11ac	0.15%	0.01%	0.00	0.96
H2AV_1_19 K7acK15ac	0.04%	0.00%	0.00	1.02
H2AV_1_19 K11acK15ac	0.09%	0.09%	0.00	0.17
H2AV_1_19 K7acK11acK15ac	0.09%	0.40%	0.00	0.80
H2AV_1_19 K4acK11acK15ac	0.00%	0.00%	0.00	1.00
H2AV_1_19 K4acK7acK15ac	0.00%	0.31%	0.00	1.00

H2AV_1_19 K4acK7acK11ac	0.05%	0.00%	0.00	1.88
H2AV_1_19 K4acK7acK11acK15ac	3.45%	4.22%	0.01	0.28
AGGKAGKDSGKAKTKAVSR(H2AZ_1_19)				
H2AZ_1_19 unmod	89.54%	89.49%	0.00	0.11
H2AZ_1_19 K4ac	1.90%	1.76%	0.00	0.20
H2AZ_1_19 K7ac	2.43%	0.59%	-0.02	4.35
H2AZ_1_19 K11ac	4.03%	4.78%	0.01	0.57
H2AZ_1_19 K15ac	1.16%	1.27%	0.00	0.62
H2AZ_1_19 K4acK7ac	0.21%	0.36%	0.00	0.74
H2AZ_1_19 K4acK11ac	0.13%	0.02%	0.00	0.93
H2AZ_1_19 K4acK15ac	0.11%	0.45%	0.00	1.37
H2AZ_1_19 K7acK11ac	0.02%	0.04%	0.00	0.70
H2AZ_1_19 K7acK15ac	0.11%	0.17%	0.00	0.60
H2AZ_1_19 K11acK15ac	0.24%	0.30%	0.00	0.30
H2AZ_1_19 K7acK11acK15ac	0.00%	0.07%	0.00	1.45
H2AZ_1_19 K4acK11acK15ac	0.00%	0.21%	0.00	1.00
H2AZ_1_19 K4acK7acK15ac	0.00%	0.48%	0.00	1.00
H2AZ_1_19 K4acK7acK11ac	0.00%	0.00%	0.00	1.00
H2AZ_1_19 K4acK7acK11acK15ac	0.13%	0.00%	0.00	1.00
AKAKTR(H2A1_12_17)				
H2A1_12_17 unmod	51.39%	33.82%	-0.18	2.02
H2A1_12_17 K13ac	0.03%	0.01%	0.00	3.20
H2A1_12_17 K15ac	25.76%	8.64%	-0.17	2.68
H2A1_12_17 K13acK15ac	0.06%	0.05%	0.00	0.69
H2A1_12_17 K15me1	13.60%	50.16%	0.37	2.86
H2A1_12_17 K13me1	9.16%	7.32%	-0.02	0.68
AKAKSR(H2A3_12_17)				
H2A3_12_17 unmod	33.21%	20.27%	-0.13	3.91
H2A3_12_17 K13ac	0.04%	0.02%	0.00	0.68
H2A3_12_17 K15ac	0.04%	0.09%	0.00	3.91
H2A3_12_17 K13acK15ac	0.08%	0.06%	0.00	0.33
H2A3_12_17 K15me1	66.07%	75.67%	0.10	5.94
H2A3_12_17 K13me1	0.57%	3.89%	0.03	2.10
DNKKTR(H2A1_72_77)				
H2A1_72_77 unmod	99.82%	97.99%	-0.02	3.26
H2A1_72_77 K74ac	0.18%	2.01%	0.02	3.26
HLQLAIR(H2A_82_88)				
H2A_82_88 unmod	100.00%	100.00%	0.00	
unmod(H2A_1_88)				
H2A_1_88 H2A14s.HLQLAIR	79.76%	81.78%	0.02	1.29
H2A_1_88 H2AZ.AGGKAGKDSGKAKTKAVSR	15.68%	13.94%	-0.02	1.58
H2A_1_88 H2AY.SAKAGVIFPVGR	0.38%	1.39%	0.01	3.02
H2A_1_88 H2AX.GKTGGKAR	4.17%	2.90%	-0.01	2.87
unmod(H2B_1_29)				
H2B_1_29 1C.PEPAKSAPAPKKGSKKAVTKAQKKDGKKR	65.57%	66.29%	0.01	0.06
H2B_1_29 1H.PDPAKSAPAPKKGSKKAVTKAQKKDGKKR	4.36%	6.84%	0.02	0.62
H2B_1_29 2F.PDPAKSAPAPKKGSKKAVTKVQKKDGKKR	2.92%	3.64%	0.01	0.24
H2B_1_29 1B.PEPSKSAPAPKKGSKKAITKAQKKDGKKR	0.03%	0.36%	0.00	2.87
H2B_1_29 1N.PEPSKSAPAPKKGSKKAVTKAQKKDGKKR	2.42%	2.73%	0.00	0.29
H2B_1_29 1D.PEPTKSAPAPKKGSKKAVTKAQKKDGKKR	17.75%	9.59%	-0.08	1.01
H2B_1_29 1M.PEPVKSAPVPKKGSKKAINKAQKKDGKKR	1.19%	0.87%	0.00	2.24
H2B_1_29 1L.PELAKSAPAPKKGSKKAVTKAQKKDGKKR	5.77%	9.67%	0.04	0.63

Table S8. Antibodies and fluorophores used for flow and microscopy studies.

**Target**	**Source**
HIV p24	MA1-7378, ThermoFisher
HSV glycoprotein B	SAB4700766, Sigma-Aldrich
Alexa Fluor 532	A20182, Invitrogen
CD3	BUV737, # 612750, BD Biosciences
CD4	BUV805, #612887, BD Biosciences
CD4	BV650, #317436, Biolegend
CXCR5	BV750, #356941; Biolegend
CD45R0	APC/Fire750, #304249, Biolegend
GATA3	PerCP/Cy5.5, #653811, Biolegend
Tbet	BV605, #644817, Biolegend
FoxP3	AF488, #320111, Biolegend
Bcl6	PE/Cy7, #358511, Biolegend
RORγT	PE, #563081, BD Biosciences

Figure S1: Infection of Jurkat T cells or primary activated CD4+ T cells with HSV-2 (333 ZAG). (A) Jurkat T cells were incubated with HSV-2 (333 ZAG), which expresses GFP from an intergenic locus under the control of the CMV promoter, at the indicated multiplicity of infection for 2 hours, washed, and cultured for a further 22 hours. The cells were stained with eFluor 450 viability dye and the percentage of live GFP positive cells quantified by flow cytometry. Data are presented as means ± SEM from two independent experiments performed in duplicate. *, $p < 0.05$; Kruskal-Wallis test. (B) Representative flow plot of activated (anti-CD3/anti-CD28) primary human CD4+ T cells infected with the GFP-expressing HSV-2 (333ZAG) at a multiplicity of infection of 1 pfu/cell.

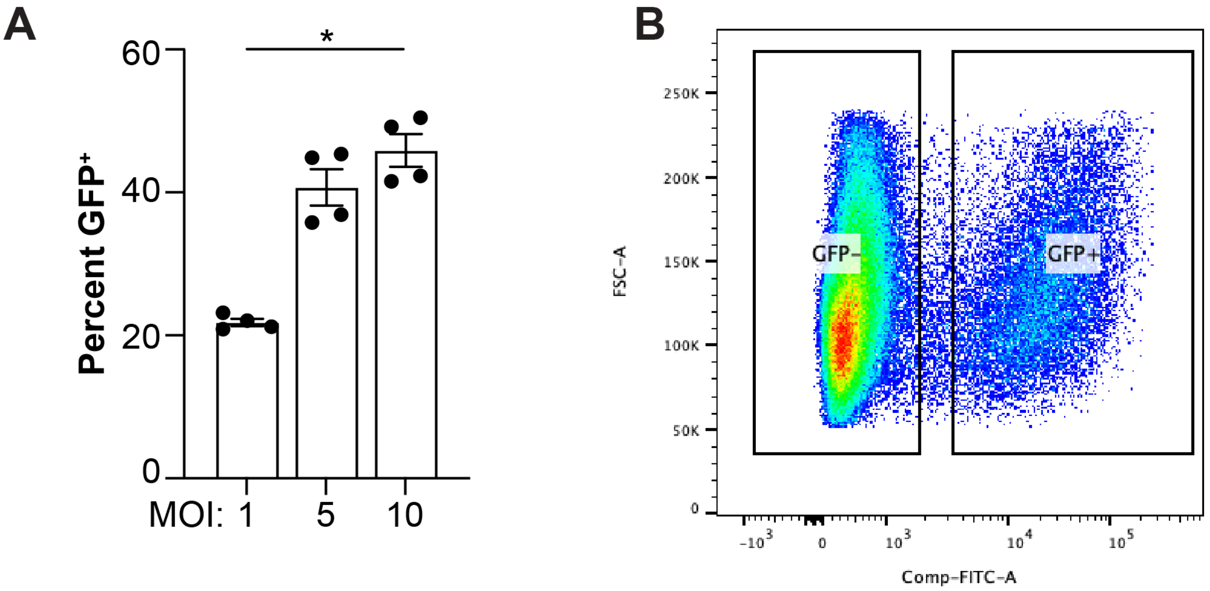

Figure S2: Expression of top 100 genes (Z-scores) contributing to principal components 1 and 2.

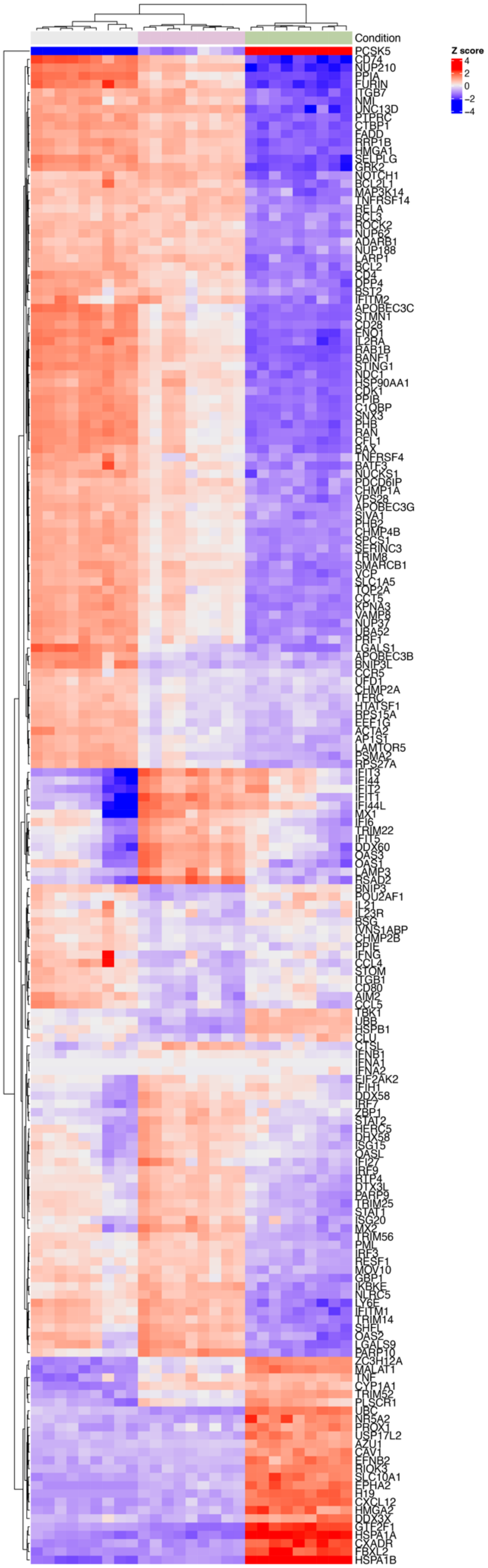

Figure S3: Single-cell RNA sequencing of HSV-2 infected activated primary tonsillar CD4+ T cells.

CD4+ T cells isolated from tonsil of an HIV-negative donor were stimulated with anti-CD3/CD28 crosslinking and then infected with HSV-2 SD90 (MOI = 1) and subjected to single-cell RNA sequencing at 0 (mock, purple), 6 (green) and 24 (red) hours post-infection.

(A) Total transcript count (library size) of cells decreases across experimental time. Values represent data following cell and gene filtering prior to normalization and denoising (see methods).

(B) Expression of UL15 per cell increases over the course of HSV-2 infection. Points represent individual cells organized by experimental timepoint.

(C) Expression of *BCL6* over the course of HSV-2 infection.

(D) Mutual information (DREMI) quantified association between expression of *UL15* and *BCL6*, *RORC* and *GATA3*.

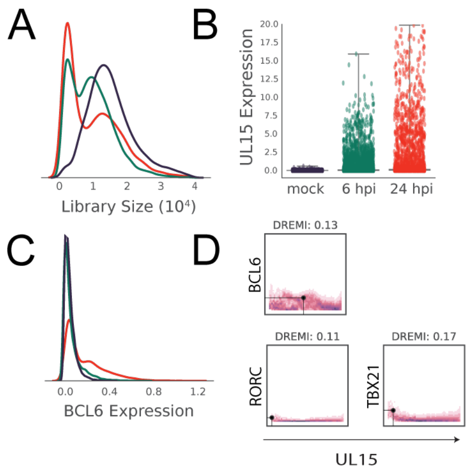

Figure S4: CRISPR/Cas9 deletion of MALAT1

(a) Gene map showing location of 5' and 3' guide RNAs and WT and KO primers. Also indicated are sites of PCR primers used for knockout confirmation. (b) Gel image of PCR products used to identify double *MALAT1* knockout (DKO, lane 5), wild-type (WT, lane 3), and heterozygotes (het) (lanes, 1, 2,4, 6, 7, and 8).

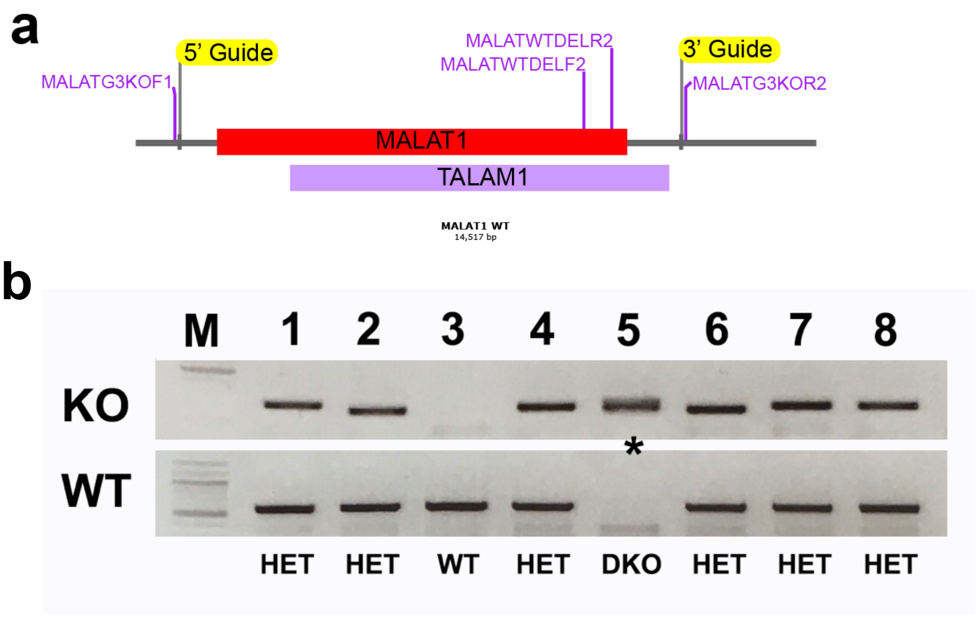

Figure S5: Representative FACS plot of VP16 transfection efficiency. 2D10 cells were transfected with 3 μg of plasmid DNA as described in the methods using program C-016. Cells were cultured for 42h following transfection prior to fluorescence activated cell sorting.

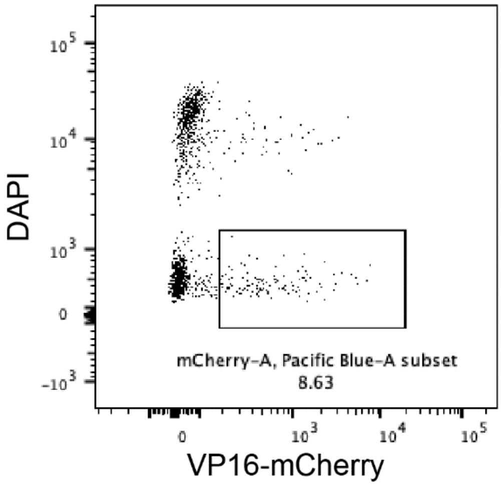

Figure S6: Proteome analysis of HSV-2 infected VP16 cells. 2D10 cells were infected with HSV-2 at a MOI of 10 pfu/cell or mock-infected and analyzed for protein expression 24 hours post-infection (n=3). Highlighted in red are HSV-2 proteins and in blue are are members of the PRC2 complex.

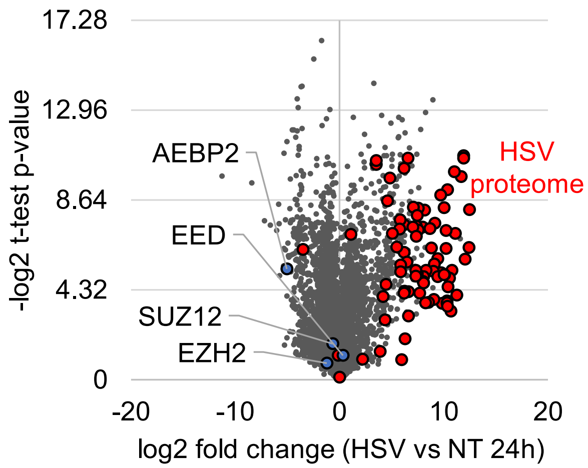

Supplement: Supplemental data [file jci-133-164317-s025.pdf]
